# Bichromatic Exon-Reporters Reveal Voltage-Gated Ca^2+^-Channel Splice–Isoform Diversity across *Drosophila* Neurons In Vivo

**DOI:** 10.1523/ENEURO.0582-24.2025

**Published:** 2025-08-14

**Authors:** Touhid Feghhi, Roberto X. Hernandez, Olena Mahneva, Carlos D. Oliva, Gregory T. Macleod

**Affiliations:** ^1^Department of Physics, Florida Atlantic University, Boca Raton, Florida 33431; ^2^Integrative Biology and Neuroscience Graduate Program, Department of Biological Sciences, Florida Atlantic University, Boca Raton, Florida 33431; ^3^International Max Planck Research School for Brain and Behavior, Jupiter, Florida 33458; ^4^Wilkes Honors College, Florida Atlantic University, Jupiter, Florida 33458; ^5^Institute for Human Health & Disease Intervention, Florida Atlantic University, Jupiter, Florida 33458; ^6^Stiles-Nicholson Brain Institute, Florida Atlantic University, Jupiter, Florida 33458; ^7^Department of Physiology, Tulane University School of Medicine, New Orleans, Louisiana 70112

**Keywords:** alternative splicing, Ca^2+^ imaging, electrophysiology, physiology, presynaptic, voltage-gated Ca^2+^ channels

## Abstract

Every neuron contains the same genomic information, but its complement of proteins is the product of countless neuron-specific steps including pre-mRNA splicing. Despite advances in RNA sequencing techniques, pre-mRNA splicing biases that favor one isoform over another are largely inscrutable in live neurons in situ. Here, in *Drosophila*, we developed bichromatic fluorescent reporters to investigate alternative splicing of *cacophony* (*cac*)—a gene that codes the pore-forming α_1_ subunit of the primary neuronal voltage-gated Ca^2+^ channel (VGCC). These reporters revealed a neuron-specific pattern of exon biases, highly consistent from one animal to the next, suggesting that each neuron splices a unique and consistent portfolio of VGCC isoforms. Stereotypical patterns were observed within motor neurons and multidendritic sensory neurons of female larvae and also within mushroom body Kenyon cells of female adults. In a validation step, we demonstrated that exon splice bias reporting was not dependent on the choice of fluorophores. Additionally, functional properties of the female larval motor neuron terminals could be generally reconciled with the functional properties predicted for the reported exon bias. The application of this technology to a large gene such as *cac* provides a precedence for effective exon-reporter design for other *Drosophila* genes.

## Significance Statement

Ca^2+^ ions are ubiquitous messengers in the nervous system, and the channels that gate their passage across membranes play a prominent role in nervous system function. In flies, as in humans, genes that code for Ca^2+^ channels give rise to different Ca^2+^ channel variants through the process of alternative gene splicing. However, at the level of individual cells in living tissues, the splicing process is largely inscrutable, making it difficult to elucidate the consequences of alternative splicing in either health or disease. Here, in the fruit fly brain, we demonstrate a technique that expresses fluorescent proteins of different colors according to the bias of the splicing process that yields different Ca^2+^ channel variants in different cells in the live animal.

## Introduction

**Figure 1.**
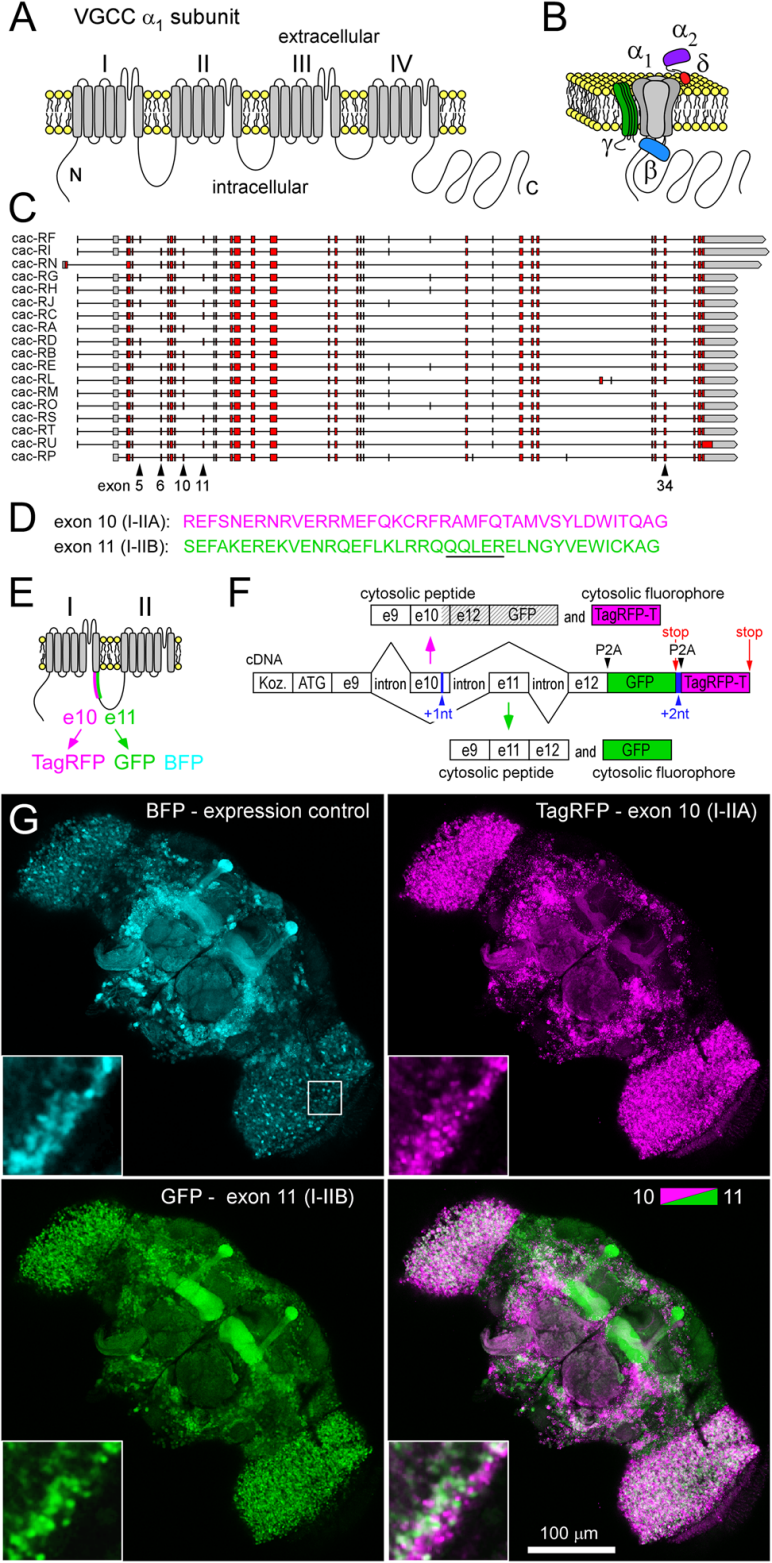
Bichromatic exon-reporters indicate biases in *cac* exon splicing between *Drosophila* neurons. ***A***, A linearized representation of the VGCC α1-pore–forming subunit (polypeptide) Cac in the plasma membrane. Homologous domains I through IV are indicated, each containing six transmembrane segments. ***B***, A 3D representation of the α1 subunit with accessory subunits β, α_2_δ, and γ. ***C***, ORF representations of *cac* splice isoforms. Two pairs of mutually exclusive exons (5 and 6 and 10 and 11) are indicated, along with the location of an exon (34) that is missing from only one of the 18 isoforms. ***D***, AA sequences determined by exon 10 (I–IIA) and 11 (I–IIB). A predicted Gβγ binding site is underlined in exon 11. ***E***, Polypeptide context for location of mutually exclusive exons 10 and 11 and their associated fluorophores, along with a third fluorophore (TagBFP) used as an independent expression control. ***F***, Schematic of DNA elements within a transgenic bichromatic exon-reporter, used to examine splicing bias of exons 10 and 11. Nucleotides are added (shown in blue) to move exons and fluorophores [GFP and TagRFP-T (TagRFP)] into frame or out-of-frame for subsequent translation, depending on pre-mRNA splicing. The nucleotide sequence is annotated in Extended Data Figure 1-1*A*. The vertical magenta and green arrows represent splicing followed by translation to yield cytosolic peptides. Hatching indicates out-of-frame peptides. ***G***, A maximum projection of a series of confocal sections (7 images, each axially separated from the next by 2 μm) through the brain of an adult. The exon 10 versus 11 reporter construct and TagBFP driven by the nSyb-GAL4 pan-neuronal driver. The brain was dissected from the head capsule prior to imaging. Scale bar, 100 μm. Inset shows detail from a single optical section on the lateral medial margin of the optic lobe (location shown by box in the BFP panel).

Voltage-gated Ca^2+^ channels (VGCCs) are essential conduits for the Ca^2+^ influx that triggers neurotransmitter release at chemical synapses and are therefore some of the most influential proteins in the nervous system. The human genome contains 10 genes for VGCC pore-forming α_1_ subunits, and a subset of these genes is expressed in individual neurons (Catterall et al., 2005). *Drosophila* has three homologs of these α_1_ subunit genes, but only one gene, *cacophony* (*cac*), represents the Ca_v_2 family of α_1_ subunits (Fig. 1*A*; Littleton and Ganetzky, 2000). However, α_1_ subunit identity alone does not determine VGCC placement and function. It is also determined by alternative splicing of the α_1_ subunit, posttranslational modifications, auxiliary subunits (Fig. 1*B*), and other associated proteins. Indeed, while *Drosophila* has only one Ca_v_2 α_1_ subunit, it has 18 annotated splice isoforms (http://flybase.org/; Fig. 1*C*). Alternative splicing confers diverse biophysical properties to Cac (Bell et al., 2025). Furthermore, Cac's roles in synapse assembly (Ghelani and Sigrist, 2018) and synaptic homeostatic plasticity (Frank et al., 2006) are also likely to depend on alternative splicing. Cell-specific information about isoform bias can give clues as to a neuron's function in the context of a circuit and even risk factors in disease processes (Lipscombe and Andrade, 2015). However, while antibodies, toxins, and pharmacological agents have been used to glean isoform-specific information, they have many limitations.

Single-cell RNA sequencing (scRNA-seq) is routinely used to transcriptionally profile individual neurons dissociated from vertebrate and invertebrate tissues, and it shows a capacity to resolve many neuron types (Zeisel et al., 2015; Davie et al., 2018). Each neuron type might be further distinguished according to differences in their “spliceosome” (Arendt-Tranholm et al., 2024; Joglekar et al., 2024), but such resolution is rarely achieved. While highly informative, scRNA-seq suffers from several limitations, such as transcriptional changes in response to the process of dissociating cells (Li, 2021) and the loss of circuit/tissue context. scRNA patch-seq, where the nuclear and cytosolic material is removed from the soma, allows for transcriptional profiling of neurons in their tissue context (Shao et al., 2023), and it has been used to identify *cac* isoforms in *Drosophila* in situ (Jetti et al., 2023). However, gaining physical access to the cell with a patch pipette requires extensive training, destroys the cell of interest, and is impractical for the purposes of assaying many neurons in the same preparation.

Here, we sought to establish a technique that would allow us to simultaneously probe exon bias in *cac* in multiple neurons in vivo. Fluorescent reporters have been used to monitor cell-specific bias in exon splicing in vivo in *C. elegans* (Kuroyanagi et al., 2006), *Drosophila* (Tadros et al., 2016; Gu et al., 2019; Li and Millard, 2019), and mouse (Takeuchi et al., 2010), but they have not been used to investigate VGCCs. These fluorescent “exon-reporters” reveal cell-specific biases in exon splicing through cell-specific differential expression of fluorophores whose expression is linked to the splicing of exons of interest, an approach previously validated through mRNA profiling in both vertebrate and invertebrate systems (Kuroyanagi et al., 2006, 2013; Oltean et al., 2008; Takeuchi et al., 2010; Li and Millard, 2019). Exon-reporter design in *Drosophila* has previously relied on modification of the gene's endogenous locus (Tadros et al., 2016; Gu et al., 2019; Li and Millard, 2019), but as the *cac* locus barely tolerates modification (Gratz et al., 2019), we developed a transgenic approach that has been particularly successful and validated in *C. elegans* (Kuroyanagi et al., 2006, 2013). This approach allowed us to map the relative distribution of *cac* exons in vivo across groups of motor and sensory neurons in the larval periphery and across mushroom body (MB) structures in the adult brain. The ability to calibrate the reporter signal, and the consistency in stereotypical signal ratios across identified sets of neurons, demonstrates the utility of bichromatic fluorescent exon-reporters for tracking gene splicing in *Drosophila*.

## Materials and Methods

### Fly stocks

*Drosophila* stocks were raised at 24°C on standard medium [Bloomington Drosophila Stock Center (BDSC) recipe]. Measurements were performed on female third instar larvae of a w^1118^ isogenized strain. BDSC provided the following fly lines: 24B-GAL4 (stock #1767) and Repo-GAL4 and nSyb-GAL4 (stock #51635). UAS-TagBFP was a gift from Dr. Kenneth Irvine.

### Fluorescent bichromatic exon-reporter design

Each construct started with a TATA box sequence followed by the standard start codon (ATG). The full DNA sequence of the *Drosophila cac* gene was obtained via FAST sequence from NCBI which was then aligned with the cDNA sequences including or lacking exons of interest using the MUltiple Sequence Comparison by Log- Expectation online tool to determine the 5′ and 3′ of exons used in construct design.

To design a construct containing two mutually exclusive exons, we included flanking introns of both exons in addition to an exon before the first mutually exclusive exon and an exon following the second mutually exclusive exon. This was done to avoid disturbing any spliceosome interactions with exons of interest and their corresponding 5′ and 3′ consensus splice sites. A reading frame of the first exon of a construct was determined by aligning an isoform's cDNA sequence with the *cac* DNA, translating the resulting exon sequence into a peptide using the Expasy online tool and using the pBLAST program to confirm the resulting peptide sequence.

The next step was to introduce one- or two-base pair addition mutations within either of the mutually exclusive exons avoiding proximity to splice sites. Introducing extra-base pairs within a spliced exon would result in a reading-frame shift downstream of that exon. An introduction of extra base pairs within a spliced exon may also result in a new stop codon, and so we avoided this by introducing several point mutations within the 3′ flanking exon of these constructs. Next, we used a 2A peptide sequence from porcine teschovirus-1 polyprotein between the 3′ exon and the first fluorophore and between the first and the second fluorophore. A GSG motif was added prior to each P2A sequence for improved cleavage of the resulting peptides. We added two base pairs after the first fluorophore to bring the second fluorophore into frame. An identical design strategy was employed for the constructs containing exons 5 and 6, as well as exons 10 and 11. A similar construct design was employed for exon 34 but without a corresponding mutually exclusive partner exon; exon 34 was flanked by 5′ and 3′ intronic material and exons 33 and 35.

The original construct containing exon 34 had two one-base-pair missense mutations including A–T substitution at the 5′ +6 position of exon 35. A *Drosophila* splice site predictor indicated that G or T are highly unlikely to appear at this position, but we checked this by designing a construct identical to the original but with an A–C point mutation to determine if the same fluorescent pattern would be observed with both constructs (see annotated sequence in Extended Data Fig. 1-1*F*). The data from the two different constructs were indistinguishable, and only data from the former construct were included in the manuscript. None of the other constructs were resynthetized due to their corresponding point mutations being at a significant distance from the 5′ or 3′ margin of exons.

The resulting DNA constructs were flanked at the 5′ and 3′ by the restriction endonuclease sites NotI and AgeI, respectively, and cloned into the multiple cloning site downstream of the hsp70 promoter in the pJFRC14 plasmid.

### Construct sequences in the order they appear in the manuscript

**Figure 2.**
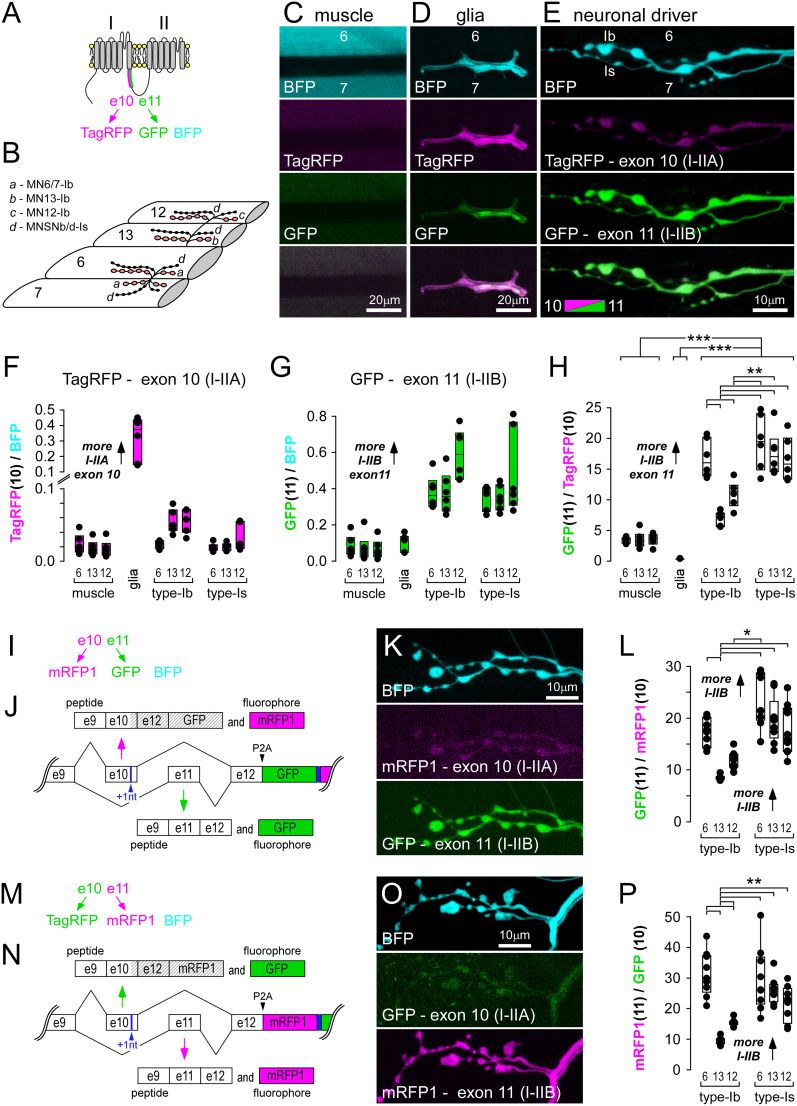
Reported splicing bias is not contingent on choice of fluorophores. ***A***, Polypeptide context for location of mutually exclusive exons 10 and 11, along with their associated fluorophores, and a TagBFP expression control. ***B***, A diagram of the general orientation of a larval filet dissection for live microscopic examination of NMJs and perineural glia on identified body-wall muscle fibers #7, 6, 13, and 12. ***C***, Muscle fibers #6 and 7, showing TagRFP and GFP expression relative to TagBFP. 24B-GAL4 driver. ***D***, Perineural glia between muscle fibers #6 and 7 showing different levels of TagRFP and GFP expression relative to TagBFP, interpreted as different biases in the inclusion of exon 10 relative to 11 in mRNA. Repo-GAL4 driver. ***E***, Presynaptic terminals of MN6/7-Ib and MNSNb/d-Is on muscle fiber #6, showing different levels of TagRFP and GFP expression relative to TagBFP. nSyb-GAL4 driver. ***F***, Fluorescence intensity of TagRFP relative to the TagBFP expression control in muscle fibers, glia, and different nerve terminals. Each point represents a different larval preparation. ***G***, Fluorescence intensity of GFP relative to the TagBFP expression control. ***H***, The ratio of GFP to TagRFP fluorescence intensity. A one-way ANOVA shows significant differences between axon terminals using Holm–Sidak post hoc tests (**p < 0.005), while a second one-way ANOVA on ranks and post hoc Dunn's method shows differences between tissue types (***p < 0.001). Boxes represent 25–75%, while the solid line marks the median. ***I***, Abbreviated logic of fluorophore association with exons. ***J***, Schematic of DNA elements within the transgenic bichromatic exon-reporter used to examine splicing bias of exon 10 (mRFP1, rather than TagRFP) versus 11 (GFP; Extended Data Fig. 1-1*B*). ***K***, Presynaptic terminals of MN6/7-Ib and MNSNb/d-Is on muscle fiber #6 expressing the construct in ***J***, and TagBFP, driven by nSyb-GAL4. ***L***, The ratio of GFP (11) to mRFP1 (10) fluorescence intensity. A one-way ANOVA shows significant differences between axon terminals using Holm–Sidak post hoc tests (asterisk, *p* < 0.005). ***M***, As in ***I*** but with exon fluorophores switched. ***N***, As in ***J*** but with exon fluorophores switched (Extended Data Fig. 1-1*C*). ***O***, Presynaptic terminals of MN6/7-Ib and MNSNb/d-Is expressing the construct in ***N***, driven by nSyb-GAL4. ***P***, The ratio of mRFP1 (11) to GFP (10) fluorescence intensity. A one-way ANOVA shows significant differences between axon terminals using Holm–Sidak post hoc tests (**p < 0.001). A regression of the GFP(10)/mRFP1(11) ratio on the reciprocal of the GFP(11)/mRFP(10) ratio (Pearson's correlation coefficient *r* = 0.968; *p* = 0.0015) is shown in Extended Data Figure 2-1.

cac_10_11_GFP_TagRFP-T (Fig. 1*F*)
cac_10_11_GFP_mRFP1 (Fig. 2*J*)
cac_10_11_mRFP1_GFP (Fig. 2*N*)
cac_5_6_GFP_mRFP1 (Fig. 4*C*)
cac_34_GFP_mRFP1 (Fig. 4*H*)
cac_34_GFP_mRFP1_remade

### Preparation of fillet-dissected larval for confocal imaging of exon-reporters

Experiments were performed on female third instar larvae. The larvae were fillet dissected in cold Schneider's insect medium on a Sylgard bath/tablet with a platform at its center, elevating the middle of the dissection. The preparation was washed thrice with cold HL3 (0.1 mM Ca^2+^, 15 mM Mg^2+^), covered with a glass coverslip, and imaged with a Nikon 60×, 1.20 NA, Plan Apochromat VC water-immersion objective on a Nikon A1R confocal microscope fitted with GaAsP detectors. Preparations were scanned sequentially, starting with the longest wavelengths and progressing to the shortest (560, 488, 405 nm). Unless indicated otherwise, images were taken using the same settings. All images represent a collapsed *Z*-series encompassing a limited depth of the ventral ganglion or the full depth of terminal boutons in the periphery (3 and 1 μm step sizes, respectively).

### Confocal imaging of exon-reporters in adult brains

We selected 1-week-old adult female flies and dissected them in cold Schneider's insect medium, where the brain, along with the attached ventral nerve cord, was carefully extracted. The preparation was then transferred to the same Sylgard bath/tablet described above with the most anterior end “propped up” on the central platform. A pin was placed over the cervical connective to hold it in place. The preparation was covered in a glass coverslip and imaged using the same settings as in larval preparations.

### Exon-reporter imaging data analysis

We analyzed the images using the ImageJ software. Measurements were obtained by calculating the average intensity from each collapsed (average) *z*-series of images. ROIs were selected using a square of 5 × 5 pixels centrally positioned on a terminal bouton, glial process, muscle fiber, neuronal cell body, or MB lobe. Care was taken to avoid the aggregates seen when using the mRFP1 fluorophore. Using Equation 8, we can quantify the molar ratio of two exons by simply multiplying the correction coefficient χ to the ratio of corresponding fluorophores. Outliers were excluded if they fell outside limits defined by 2.5 times the median absolute deviation.

### Electron microscopy

The number of AZs (N_AZ_) at each terminal was determined through a combination of light microscopy estimates of terminal volume published previously (Table 1-1 of the Extended Data Set of Justs et al., 2022; listed here in Column 2 of Table 2) and transmission electron microscopy estimates of the number of AZs per unit terminal volume. Five series of micrographs (100 nm sections), each from a separate larva and previously described in both Lu et al. (2016) and Justs et al. (2022), were analyzed to estimate the number of AZ per unit volume (Column 3 in Table 2). The Type Is terminals could be distinguished from Type Ib without ambiguity through reference to synaptic vesicle (SV) outer diameter (type Is, 43.97 ± 0.05 nm; Type Ib, 33.67 ± 0.05 nm; mean ± SEM), and each Type Ib MN could be distinguished from another without ambiguity through reference to muscle fiber identity. The minimum terminal volume sampled in each series was >1 μm^3^ for Type Is and >5 μm^3^ for Type Ib. Finally, N_AZ_ was determined by multiplying average terminal volume by the average number of AZs per unit volume (Column 4 in Table 2).

### Electrophysiology

Two-electrode voltage clamp (TEVC) was used to quantify terminal-specific release on each of body-wall muscle fibers #6, 13, and 12. Electrophysiology was conducted on female third instar larvae, from an in-house *w*^1118^ wild-type stock, in hemolymph-like solution #6 (HL6; Macleod et al., 2002) containing MgCl_2_ added to 15 mM and CaCl_2_ added to 2 mM. Fillet dissections were performed in chilled HL6 on Sylgard plates, and recordings were made 20–60 min after transecting the segmental nerves. Signals were detected, digitized, and recorded using an Axoclamp 900A amplifier (Molecular Devices) connected to a 4/35 PowerLab (ADInstruments) and a PC-running LabChart v8.0. Micropipettes filled with a 1:1 mixture of 3 M KCl and 3 M K-acetate. Measurements were performed on segment #4 using a BX50WI Olympus microscope and a 20× water-dipping objective to allow unequivocal identification of muscle fibers. Recordings commenced in a current-clamp mode, with two different micropipettes in two different muscle fibers. A suction pipette applied 0.3 ms electrical impulses to the transected nerve to evoke release from MN terminals. Impulse voltage was incrementally increased to initiate APs in one MN but not the other, and knowledge of the stereotypical innervation was relied upon to determine the identity of the MN terminal responsible for evoked release (Lu et al., 2016). Once stimulus thresholds were established, one micropipette was removed and placed in the same muscle fiber as the other micropipette and TEVC was initiated. A minimum of 10 excitatory junctional currents (EJCs) were recorded during 0.2 Hz stimulation, along with 30 miniature EJCs (mEJCs), from each muscle fiber. Recordings of EJCs were made from 15 MN6-Ib terminals, 4 MN13-Ib, 24 MN12-Ib, 20 MNSNb/d-Is M#6, 9 MNSNb/d-Is M#13, and 6 MNSNb/d-Is M#12, and mEJCs were recorded from 58 #6, 16 #13, and 61 #12 muscle fibers.

Quantal content (QC) was calculated by dividing the mean EJC amplitude by the corrected mean mEJC amplitude. A correction factor must be applied to the mean mEJC amplitude to obtain a better estimate of terminal-specific QC. The correction is needed as the identity of the MN responsible for mEJCs is inscrutable using TEVC, yet mEJCs originating from Type Is terminals are 50% larger than those originating from Type Ib terminals (Karunanithi et al., 2002; Pawlu et al., 2004; Dawson-Scully et al., 2007; Han et al., 2022). As each terminal is responsible for a similar number of spontaneous events, a correction factor of 0.8 is applied to the mean mEJC amplitude (i.e., reduced by 20%) when calculating QC for Type Ib terminals, and a correction factor of 1.2 used for Type Is terminals (i.e., increased by 20%; Lu et al., 2016). The average probability of release at individual active zones (AZs; P_AZ_; Table 2, Column 6) was calculated as the QC for a single isolated action potential (AP; QC; Justs et al., 2022; column 5, Table 2) divided by the average number of AZs (N_AZ_; column 4, Table 2).

Motor neuron endogenous firing frequencies were determined in 3rd instar larvae during fictive locomotion as described by Chouhan et al. (2010, 2012). The firing frequencies for MN13-Ib and MNSNb/d-Is M#13 were determined by Chouhan et al. (2010), while those of MN6-Ib, MN13-Ib, MN12-Ib were determined by Chouhan et al. (2012).

### Ca^2+^ imaging and estimation of Ca^2+^ entry

Justs and others reported the number of calcium ions (Ca^2+^) that enter each of the terminals with each AP (Table 3-1 of the Extended Data Set of Justs et al., 2022; listed here in Column 7 of Table 2). All details of Ca^2+^ imaging and analysis are described by Justs et al. (2022). They are based on Ca^2+^ transients quantified using the dextran-conjugated Ca^2+^ indicator (rhod) loaded in consistent proportion to a dextran-conjugated Ca^2+^ insensitive dye (AF647). Fluorescence responses to single AP-triggered Ca^2+^ transients were collected on EMCCD cameras running at 100 fps, while the terminals were bathed in HL6 saline (2 mM Ca^2+^ and 15 mM Mg^2+^). We divided the number of Ca^2+^ entering a terminal by the total number of AZs in that terminal (N_AZ_; Table 2, Column 4) to calculate the terminal-specific average number of Ca^2+^ entering through an individual AZ during an AP (Ca^2+^_AZ_; Table 2, Column 8). Values of Ca^2+^_AZ_ are intended to represent Ca^2+^ entry through Cac VGCCs, but a substantial amount of Ca^2+^ also enters through VGCCs built around another α_1_ subunit (DmCA1D; Krick et al., 2021), and so Ca^2+^_AZ_ values must be considered overestimates. The true Ca^2+^_AZ_ will vary in proportion to these overestimates to the extent that Cac and DmCa1D are present in the same proportion in the different terminal types. In recognition of these limitations, the absolute average number of Ca^2+^ entering through an individual AZ during an AP cannot be indicated on the ordinates in Figure 6, *F*, *J*, and *N*.

### Statistical analysis and data presentation

Statistical tests were performed using SigmaStat 3.5 (integrated with SigmaPlot 10). Significance was assessed with an *α* of <0.05. Student's *t* tests were used for comparisons between two populations and Mann–Whitney rank-sum tests were used as a nonparametric alternative. If more than one test was applied, *α* was adjusted accordingly using Bonferroni’s correction. Where analysis of variance (ANOVA) was used for multiple comparisons, an overall α of <0.05 was required to claim significance. ANOVAs were run on ranks when tests for data normalcy failed. Propagation of uncertainty theory (Farrance and Frenkel, 2012) was used to calculate variance of means based on uncertainty measurements combined from different techniques. Pearson's product–moment correlation coefficient was calculated to test the strength and direction of associations. The ordinary least-squares method was used to provide linear fits.

## Results

### A bichromatic exon-reporter reveals splicing bias between *Drosophila* neurons

We initially probed splicing of Exons 10 and 11 of *cac*, mutually exclusive exons which code for alternative peptides that form the 5′ extent (40AAs) of the intracellular loop linking homologous domains I and II of the α_1_ subunit (I–IIA and I–IIB, respectively; http://flybase.org/; Peixoto et al., 1997; Bell et al., 2025; Fig. 1*D*,*E*). Transgenic bichromatic exon-reporters are composed of DNA that mimics a limited stretch of a gene's open reading frame (ORF) along with DNA sequences encoding two distinct fluorophores (Fig. 1*F*; Extended Data Fig. 1-1*A*). The gene's ORF contains the exons of interest and, in an attempt to preserve relevant splice sites, a minimum of a flanking exon and a flanking intron on each side (see Materials and Methods). DNA of the exon-reporter was integrated into the fruit fly's second chromosome, and a GAL4-responsive upstream activation sequence (UAS) allowed for its conditional expression. Spliceosomes will process pre-mRNA transcribed from both the gene's endogenous locus and the exon-reporter transgene. If the spliceosome retains exon 10, but excises exon 11, this construct will express TagRFP-T (TagRFP; Fig. 1*F*). Retaining exon 11 while excising exon 10 will result in EGFP (GFP) expression. An essential element of this design is to place GFP and TagRFP in different “frames,” and the frame is then determined by pre-mRNA splicing. This design will also yield peptides corresponding to exons and fluorophores translated both in-frame and out-of-frame. The potential for deleterious effects arising from competitive peptides is considered in a later section. TagBFP expression from a standard UAS construct was used as an expression control.

10.1523/ENEURO.0582-24.2025.f1-1 Figure 1-1 Exon reporter construct nucleotide sequences. The nucleotide sequence of each exon reporter has been annotated to allow substantiation of the intended effect of adding nucleotides to change frame. A. cac_10_11_GFP_TagRFP-TB. cac_10_11_GFP_mRFP1C. cac_10_11_mRFP1_GFPD. cac_5_6_GFP_mRFP1E. cac_34_GFP_mRFP1F. cac_34_GFP_mRFP1_remade Download Figure 1-1, DOCX file.

**Figure 3.**
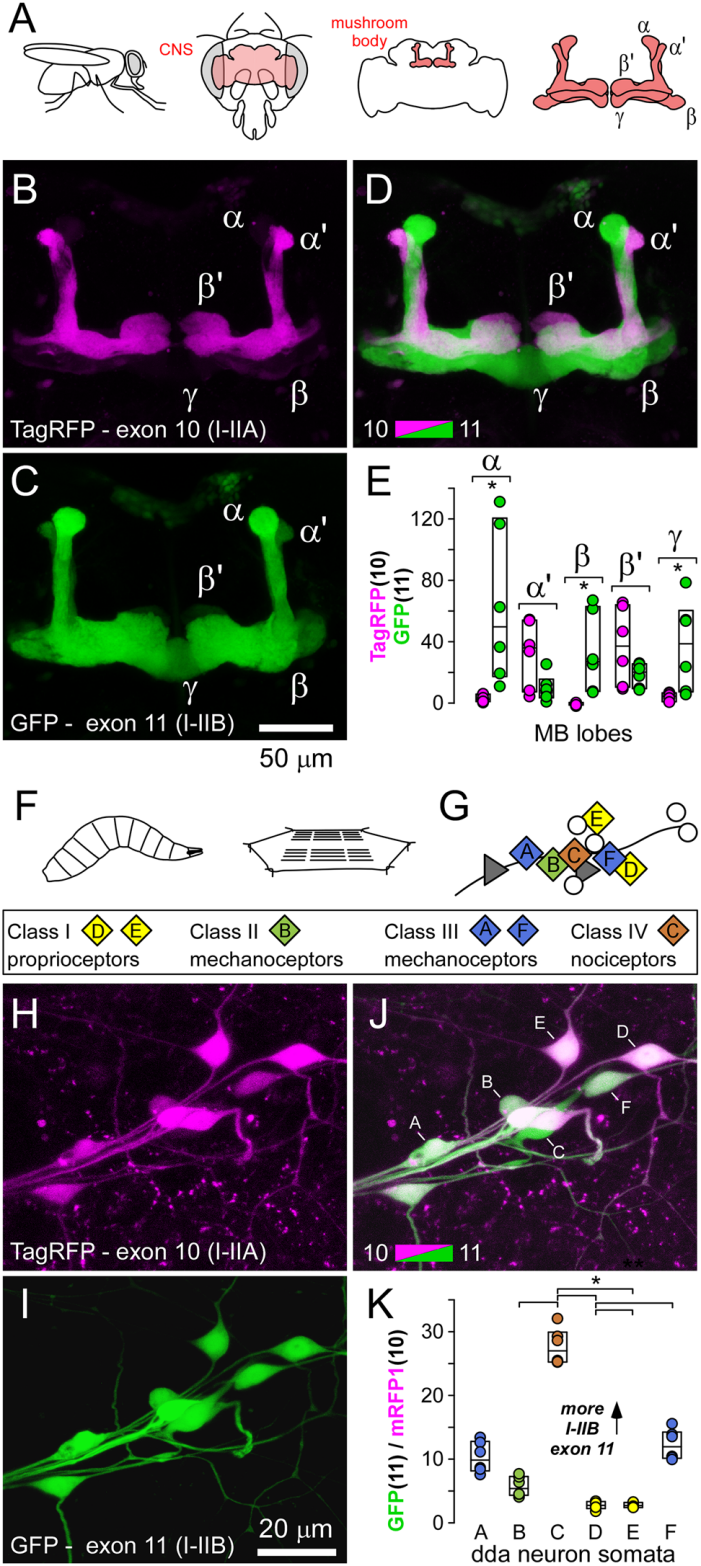
Stereotypical differences between MB structures and sensory neurons. ***A***, A diagram of the orientation of the MB and its lobes, within the brain, and head capsule of the adult fruit fly. ***B–D***, A MB, with lobes identified, with the exon 11 (GFP) versus 10 (TagRFP) reporter construct driven by the nSyb-GAL4 pan-neuronal driver. ***E***, The fluorescence intensity of GFP and TagRFP in the different lobes. Ratios were not plotted as faint lobular expression sometimes gave negative fluorescence values after background subtraction. Significant differences (*p* < 0.01) in intensity are indicated with an asterisk (Mann–Whitney rank-sum tests; α adjusted to 0.01 after Bonferroni’s correction for 5 tests). ***F***, A diagram of the general orientation of a larval filet dissection for live examination of da neurons, sensory neurons that form multiple branched dendrites on the larval epidermis between the cuticle and body-wall muscles. ***G***, The somata of four different morphological classes of da neurons can be stereotypically identified in a dorsal cluster in each abdominal hemisegment (after Grueber and others; Grueber et al., 2003); ddaA through ddaF as shown. ***H***, ***J***, A dorsal cluster of sensory neuron somata with the exon 10 (TagRFP) versus 11 (GFP) reporter construct driven by the nSyb-GAL4 pan-neuronal driver. ***K***, The ratio of GFP to TagRFP fluorescence intensity. A one-way ANOVA on ranks shows significant differences between axon terminals using Tukey pairwise post hoc tests (asterisk, *p* < 0.001).

In an initial test of the utility of this construct, we drove expression pan-neuronally and observed stark differences in fluorescence intensities of the two fluorophores in the live brain of adult flies (Fig.1*G*). GFP fluorescence was particularly bright relative to TagRFP fluorescence in most lobes of the MB (investigated further in Fig. 3), and differences in fluorophore expression were evident between neurons within the optic lobes (Fig. 1*G*, inset). We interpreted this pattern as indicating differences in the splicing bias between neurons in the MB and neurons in the optic lobes.

### Exon-reporter expression levels vary across tissue types

To better interpret fluorophore expression patterns, we examined fluorescence intensities at the level of individual cells, which are readily identifiable at the larval neuromuscular junction (NMJ). Established GAL4 drivers were used to drive the reporter construct for exon 10 versus 11 (Fig. 2*A*) in the muscle (24B), glia (Repo), and motor neurons (MNs; nSyb). A total of three glutamatergic MNs innervate muscle fibers #7, 6, 13 and 12 with large bouton terminals [Type Ib (big); Fig. 2*B*]; MN6/7-Ib forms a NMJ across muscle fibers #7 and 6, MN13-Ib forms a NMJ on fiber #13, and MN12-Ib forms a NMJ on fiber #12. A fourth glutamatergic MN (MNSNb/d-Is) forms a NMJ with small bouton terminals [Type Is (small)] on each of muscle fibers #7, 6, 13, and 12. Construct expression in muscle fibers resulted in low levels of GFP and TagRFP relative to the expression control (Fig. 2*C*), suggesting a low level of *cac* pre-mRNA splicing and functional VGCCs, as functional VGCCs require either exon 10 or exon 11. Expression in glia resulted in weak GFP expression but very strong TagRFP expression (Fig. 2*D*), suggesting that Repo-positive perineural glia may use Cac VGCC isoforms that rely on exon 10. Expression in MNs revealed a higher level of GFP than TagRFP (Fig. 2*E*), in all MN terminals, indicating a splicing bias in favor of exon 11 (I–IIB; Fig. 2*F–H*). Interestingly, there were significant differences in the ratio of GFP to TagRFP fluorescence between Type Ib axon terminals that belong to different MNs. However, the ratio of GFP to TagRFP fluorescence was no different between Type Is terminals that all originate from an axon of a single MN (MNSNb/d-Is; Fig. 2*H*).

10.1523/ENEURO.0582-24.2025.f2-1 Figure 2-1 **Correlation between two versions of an exon 10 versus 11 reporter with fluorophore construct order reversed.** Terminal ratios obtained from exon reporter GFP (10) / mRFP1 (11) (shown in Fig. 2P) are plotted against the inverse of terminal ratios from exon reporter mRFP1 (10) / GFP (11) (shown in Fig. 2L). Pearson’s correlation coefficient indicated a significant correlation (R^2^ = 0.937, P = 0.0015). Download Figure 2-1, TIF file.

The pan-neuronal driver nSyb-Gal4 also drives expression in the Type II monoaminergic terminals and Type III peptidergic terminals, and we confirmed that exon-reporter signals were quite strong in Type II terminals (e.g., Extended Data Fig. 4-1). This might have been expected as Type II terminals also express Cac VGCCs and traffic them to Brp-defined AZs. We were unable to definitively identify Type III terminals on muscle fiber #12. Throughout this study, we focused on Type Ib and Is terminals, excluding Type II and III, as only Type Ib and Is are readily tractable for physiological analyses.

### Reporting of splicing bias is not dependent on the choice of fluorophore

To determine whether differences in the ratio of GFP to TagRFP might be a function of different rates of protein maturation or degradation in different neurons, rather than differences in splicing per se, we reversed the order of fluorophores in the construct and quantified the ratios (Fig. 2*I*–*P*). Unfortunately, TagRFP, which rarely forms aggregates, could not be used as it could not be placed one or two nucleotides out-of-frame without the creation of premature stop codons. Since mRFP1 does not generate stop codons when out-of-frame, it was paired with GFP to report the presence of exon 10 versus 11 (Fig. 2*I*–*L*; Extended Data Fig. 1-1*B*) and vice versa (Fig. 2*M*–*P*; Extended Data Fig. 1-1*C*). With mRFP1 in place of TagRFP, the reporter construct duplicated the pattern of exon bias toward exon 11 reported by the original TagRFP/GFP combination (compare Fig. 2*L* with Fig. 2*H*). Furthermore, the order of the fluorophores made little difference to the estimated ratio of exon 10 versus 11 when using the same microscopy settings for the different transgenes, i.e., the ratios in Figure 2*P* show a similar pattern to the ratios in Figure 2*L*, and the regression of one on the other showed a significant positive correlation (Extended Data Fig. 2-1; *r* = 0.968; *p* = 0.0015).

### Bichromatic exon-reporters can be calibrated

Bichromatic exon-reporters can be calibrated to ensure accurate representation of splicing ratios. Reversal of the fluorophore order also provides an opportunity to calibrate the ratio of fluorescence intensities in terms of a molar ratio, i.e., the relative number of fluorescent proteins. This approach allows us to nullify the effect of differences in microscope settings used for the different fluorophores. The fluorescence intensity f measured in each experiment can be expressed as follows:

f=n*QY*s,

where *n* is the number of fluorophore proteins corresponding to the exon it represents, QY is the quantum yield of the fluorophore, and s represents the effect of microscope settings particular to that fluorophore, such as collection efficiency (depends on the numerical aperture of the objective lens and other optical elements), power of the excitation laser, gain of the photomultiplier, and extinction coefficient of GFP or mRFP1 at the excitation wavelength.

Using the exon 10 GFP and exon 11 mRFP1 construct, the fluorophore number n can be expressed as follows:

$$n_{10}=\frac{\;f_{\mathrm{GFP},10}}{QY_{\mathrm{GFP}}\;*\;S_{\mathrm{GFP}}}.$$

If we reverse the fluorophores such that mRFP1 reports exon 10, GFP reports exon 11, and $f_{\mathrm{GFP},11}^{*}\;$ shows fluorescence intensity for the reverse construct, we can similarly write as follows:

$$n_{11}=\frac{\;f_{\mathrm{GFP},11}^{*}\;}{QY_{\mathrm{GFP}}\;*\;S_{\mathrm{GFP}}}.$$

Combining Equations 2 and 3 gives the following:

$$\frac{n_{10}}{n_{11}}=\frac{\;f_{\mathrm{GFP},10}}{\;f_{\mathrm{GFP},11}^{*}\;}.$$

We can derive similar equation using mRFP1 instead of GFP:

$$\frac{n_{10}}{n_{11}}=\frac{\;f_{\mathrm{mRFP}1,10}^{*}\;}{\;f_{\mathrm{mRFP}1,11}}.$$

Alternatively, the ratio of proteins can be calculated using Equation 6:

$$\frac{n_{10}}{n_{11}}=\left(\frac{QY_{\mathrm{mRFP}1}\;*\;S_{\mathrm{mRFP}1}}{QY_{\mathrm{GFP}}\;*\;S_{\mathrm{GFP}}}\right)\;*\;\;\frac{\;f_{\mathrm{GFP},10}}{\;f_{\mathrm{mRFP}1,11}}.$$

$\mathrm{The}\;\mathrm{unknown}\;\mathrm{factor}\;\chi =\left(\frac{QY_{\mathrm{mRFP}1}\;*\;S_{\mathrm{mRFP}1}}{QY_{\mathrm{GFP}}\;*\;S_{\mathrm{GFP}}}\right)$ appearing in Equation 6 can be calculated using Equations 4–6 as follows:

$$\chi =\sqrt{\frac{\;f_{\mathrm{mRFP}1,11}}{\;f_{\mathrm{GFP},10}}\;*\;\frac{\;f_{\mathrm{mRFP}1,10}^{*}\;}{\;f_{\mathrm{GFP},11}^{*}\;}}.$$

For the selected fluorophores and microscope settings, we calculated the value of χ across different neurons and muscle fibers (Table 1). For two terminal types (1b and 1s) across three different muscle fibers (#6, 13, and 12), the average value of χ is 1.12, and $CV^{2}$ of χ is 0.05. This low $CV^{2}$ indicates that the bichromatic exon-reporters yield the same apparent ratio of splicing bias across different contexts. Knowing this value, the molar ratio of fluorophores representing the splicing bias can be calculated by multiplying the ratio of their fluorescence intensities by χ or as follows:

$$\frac{n_{10}}{n_{11}}=\chi \;*\;\;\frac{\;f_{\mathrm{GFP},10}}{\;f_{\mathrm{mRFP}1,11}}.$$

**Table 1.** Values of the average correction factor (*χ*) and the square of its coefficient of variation (CV^2^)

Terminal identity	Value of χ(Eq. 7; ±SD)	CV^2^
MN6/7-Ib	1.294 ± 0.242	0.035
MN13-Ib	1.051 ± 0.153	0.021
MN12-Ib	1.010 ± 0.207	0.042
MNSNb/d-Is, M6	1.007 ± 0.238	0.056
MNSNb/d-Is, M13	1.201 ± 0.374	0.097
MNSNb/d-Is, M12	1.072 ± 0.255	0.057

Our conclusion is that, through serendipity alone, the relative values of fluorescence intensities closely represent the relative numbers of fluorophores.

### Exon-reporters reveal differences between MB structures and peripheral sensory neurons

The reporter constructs for exon 10 versus 11 also revealed stereotypical and highly consistent reporter ratios between lobes of the adult MB and peripheral sensory neurons of larvae (Fig. 3). The MB structure has been annotated according to the regions where cholinergic Kenyon cells (KCs) of the calyx extend their axons, and it is divided into a number of overlapping lobes (α, α′, β, β′ and γ; Crittenden et al., 1998; Fig. 3*A*). In vivo examination revealed α′ and β′ lobes to be characterized by TagRFP fluorescence, representing exon 10 (I–IIA), while α, β, and γ lobes were characterized by GFP fluorescence, representing exon 11 (I–IIB; Fig. 3*B*–*D*). This pattern was highly consistent across multiple flies (Fig. 3*E*), suggesting that KCs contributing axons to α′ and β′ lobes primarily rely on VGCC α1 subunit isoforms using exon 10, represented by Cac-RA, Cac-RB, Cac-RE, Cac-RH, Cac-RI, Cac-RL, Cac-RM, Cac-RN, Cac-RO, and Cac-RP, while cells contributing axons to α, β, and γ lobes use exon 11, represented by Cac-RC, Cac-RD, Cac-RF, Cac-RG, Cac-RJ, Cac-RS, Cac-RT, and Cac-RU. Greater detail of splicing bias indicated by the exon-reporter can be seen in the single sections of a high magnification confocal series (Movie 1).

**Movie 1.** A series of confocal sections through a live mushroom body-rendered as a movie. A series of confocal sections (12 images, each axially separated from the next by 2 μm) through MB lobes of an adult fly. The exon 10 (TagRFP) versus 11 (GFP) reporter construct and TagBFP were driven by the nSyb-GAL4 pan-neuronal driver. The brain was dissected from the head capsule prior to imaging. Frame dimensions are 133 × 133 μm. [View online]

The same reporter construct was examined in a dorsal cluster of dendritic arborization (da) neurons. These sensory neurons form multiple branched dendrites on the basal surface of the epidermis between the cuticle and body-wall muscles (Fig, 3*F*–*K*). The somata of six da neurons, representing four different morphological classes, can be stereotypically identified in each abdominal hemisegment (ddaA-F; Fig. 3*G*; Grueber et al., 2002). Here, we again observed strong stereotypical differences in fluorophore expression and presumably exon bias (Fig. 3*H–K*) where exon 11 (I–IIB) is favored in all da neurons. Class category (I, II, III, and IV) increases with increasing territory size and/or branching complexity (Grueber et al., 2002), and intriguingly, the ratio of exon 11 to 10 also increases with class category; Class I (D and E) < Class II (B) < Class III (A and F) < Class IV (C; Fig. 3*K*).

### Exon-reporters reveal consistent differences in splicing bias of other exons

**Figure 4.**
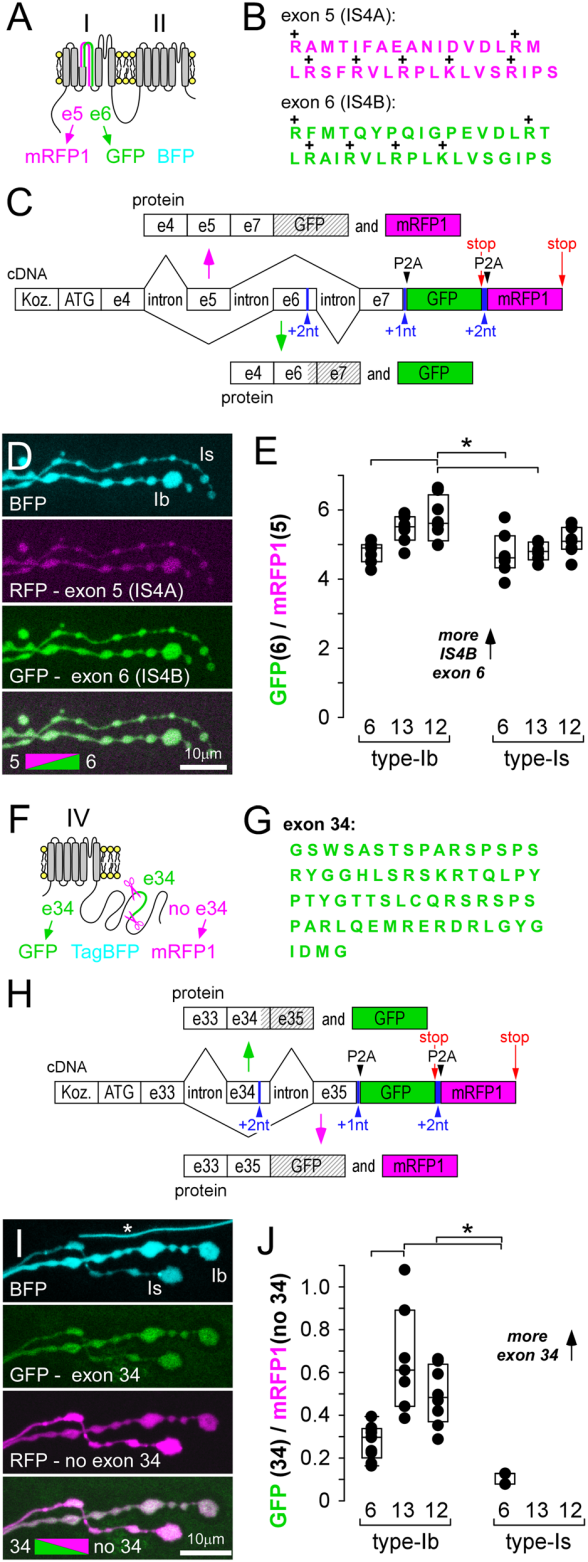
Exon-reporters for exons 5 versus 6, and 34, also show stereotypical patterns of bias. ***A***, Protein context for location of mutually exclusive exons 5 and 6, their associated fluorophores, and a TagBFP expression control. ***B***, AA sequences determined by exons 5 and 6. ***C***, Schematic of DNA elements within a transgenic bichromatic exon-reporter, used to examine splicing bias of exons 5 and 6 (see Extended Data Fig. 1-1*D*). ***D***, Presynaptic terminals of MN6/7-Ib and MNSNb/d-Is on muscle fiber #6, showing mRFP1 and GFP expression relative to TagBFP. nSyb-GAL4 driver. ***E***, The ratio of GFP to mRFP1 fluorescence intensity. A one-way ANOVA shows significant differences between axon terminals using Holm–Sidak post hoc tests (*p < 0.005). ***F***, Protein context for location of exon 34, the associated fluorophores, and a TagBFP expression control. ***G***, AA sequences determined by exon 34. ***H***, Schematic of DNA elements within a transgenic bichromatic exon-reporter, used to examine splicing bias of exon 34 (Extended Data Fig. 1-1*E,F*). ***I***, Presynaptic terminals of MN6/7-Ib and MNSNb/d-Is on muscle fiber #6, showing GFP and mRFP1 expression relative to TagBFP. Asterisk denotes autofluorescence from a fine tracheole. nSyb-GAL4 driver. ***J***, The ratio of GFP (includes exon 34) to mRFP1 (exon 34 excised) fluorescence intensity. A one-way ANOVA shows significant differences between axon terminals using Holm–Sidak post hoc tests (*p < 0.005). MNSNb/d-Is terminals were often missing or difficult to identify on muscles fibers #13 and 12 (Extended Data Fig. 4-1).

Bichromatic exon-reporters can be used to interrogate splicing of other mutually exclusive exons (5 vs 6), as well as a seemingly nonessential exon (34; Lembke et al., 2019; Fig. 4). Exons 5 and 6 each determine the identity of two different peptides of 35AAs which form the S4 voltage sensor in the first homologous domain (IS4A and IS4B, respectively; http://flybase.org/; Peixoto et al., 1997; Bell et al., 2025; Fig. 4*A*,*B*). The ratio of fluorophores corresponding to exon 5 (mRFP1) versus exon 6 (GFP; Fig. 4*C*; Extended Data Fig. 1-1*D*) was consistent between MNs from one larva to the next (Fig. 4*D*,*E*), just as it was with mutually exclusive exon 10 versus 11 (Fig. 2). The ratios suggest that MNs rely most heavily on VGCC α1 subunits using exon 6 (IS4B), an exon that carries less positive charge in the S4 voltage sensor compared with the alternative exon (5; IS4A).

10.1523/ENEURO.0582-24.2025.f4-1 Figure 4-1 **Type-Is terminals missing phenotype observed when expressing the exon 34 reporter.** A-B. Muscle fibers #6 and #7 showing the TagBFP expression control, along with GFP and mRFP1 of the exon 34 reporter when expressed pan-neuronally using the nSyb-GAL4 driver. Type-Ib terminals can be clearly identified, while type-Is terminals appear “vestigial” in some cases (A), if not missing (B). This phenotype was observed in two of the 9 preparations examined. C-E. Muscle fibers #13 and #12 showing the TagBFP expression control, along with the exon 34 reporter, when expressed with the nSyb-GAL4 driver. An attempt is made to identify each terminal type, but that cannot be done definitively when one terminal is missing (C & D: type III missing), or more than one terminal is missing (E: type-II and type III missing), on one or both muscle fibers. Download Figure 4-1, TIF file.

Exon 34 determines the identity of 68AAs in the middle of the carboxy terminus in all but one Cac isoform (Cac-RM; http://flybase.org/; Chang et al., 2014; Lembke et al., 2017, 2019; Figs. 1*C*, 4*F*–*G*; Extended Data Fig. 1-1*E*). Surprisingly, we found that the fluorophore corresponding to splicing exon 34 out of the pre-mRNA (mRFP1) was present in all neurons and present at higher levels than the fluorophore corresponding to the presence of exon 34 (GFP), indicating high levels of the Cac-RM isoform in all cells. The ratio reported in MNSNb/d-Is terminals indicated that these terminals had the lowest proportion of Cac isoforms with exon 34, but the same terminals were often physically missing (Extended Data Fig. 4-1).

### Exon-reporters reveal differences between neurons in the larval ventral ganglion

**Figure 5.**
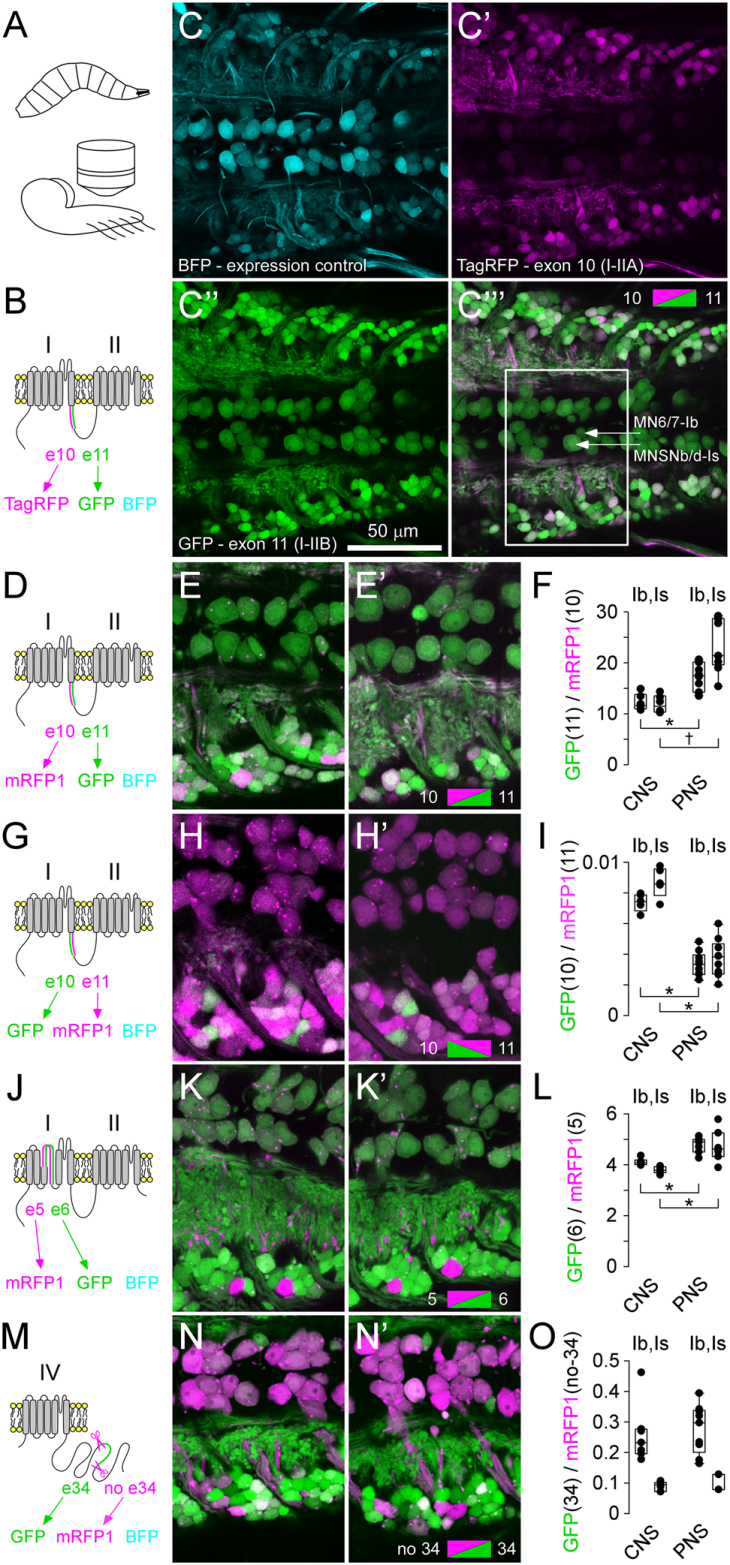
Stereotypical patterns of exon bias in the larval ventral ganglion. ***A***, A diagram of the general orientation of a larval ventral ganglion preparation for live microscopic examination. ***B***, Polypeptide context for location of mutually exclusive exons 10 and 11 and a TagBFP expression control. ***C–C*’’’**, Neurons in the larval ventral ganglion (VG) expressing the exon 10 (TagRFP) versus 11 (GFP) reporter construct and a TagBFP expression control. ***D***, ***G***, ***J***, ***M***, Polypeptide context for location of probed exons—data grouped in rows. ***E***, ***E*’**, ***H***, ***H*’**, ***K***, ***K*’**, ***N***, ***N*’**, Fields-of-view, similar in extent to the solid line inset in ***C***, spanning Hemisegments 3 and 4. Two separate larval VGs are shown in each of ***E***, ***E*’**, ***H***, ***H*’**, ***K***, ***K*’**, ***N***, and ***N*’**. In each case, the nSyb-GAL4 driver was used, and a single confocal section captured most of the dorsal midline MN somata. ***F***, ***I***, ***L***, ***O***, The fluorophore ratio intensity measurements for each reporter, shown immediately to the left, for somata of MN6/7-Ib and MNSNb/d-Is in the central nervous system (CNS), and for terminals of the same MN type in the periphery (PNS). CNS location of the somata shown in inset in ***C***. Asterisks indicate significant differences (*p* < 0.005) in Student's *t* tests. α adjusted to 0.025 after Bonferroni’s correction for two tests. The dagger indicates a significant difference (*p* = 0.002) in a Mann–Whitney rank-sum test.

To determine the extent to which larval central neurons might manifest differences in exon bias, we drove expression with a pan-neuronal driver (nSyb) and examined patterns within the ventral ganglion (Fig. 5). In vivo examination revealed diverse but highly consistent patterns from one larva to the next. The consistency is demonstrated by the common patterns observed across different larvae for each reporter construct (Fig. 5*B*,*D*,*G*,*J*,*M*). The patterns were consistent when different fluorophores were used, i.e., mRFP1 used in place of TagRFP for mutually exclusive exons 10 and 11 (compare solid line inset of Fig. 5*C*,*E*), and the pattern inverted when the fluorophores were reversed (compare Fig. 5*E*,*H*).

### Exon-reporters suggest subcellular differences in exon bias

To determine whether the pattern in the somata represents the same pattern observed in the axon terminals of the same MN type, we compared the central values with the peripheral values (Fig. 5*F*,*I*,*L*,*O*). Reporter ratios were generally preserved but the bias toward exon 11 (I–IIB) was stronger in the terminals compared with somata in both terminal types (Fig. 5*F*). This was unexpected, as pre-mRNA splicing typically occurs in the nucleus and we anticipated a constant ratio across all parts of the neuron if GFP diffuses as readily as mRFP1. A P2A peptide sequence between exon-associated peptides and the fluorophore ensures that the fluorophore is free to diffuse unencumbered by exon peptides. When fluorophores were reversed, relative to the exons (Fig. 2*N* vs *J*), the greater bias toward exon 11 in the terminals was preserved (compare Fig. 5*I* with *F*). A greater bias toward exon 6 (IS4B), relative to exon 5, was also observed in terminals compared with their somata (Fig. 5*L*). We observed no difference in biases toward constructs containing exon 34 in terminals relative to somata (Fig. 5*O*).

### In vivo presynaptic properties are consistent with exon-reporter ratios

To determine whether the fluorophore ratios established here do indeed represent a splicing bias and presumably the mRNA ratio of *cac* splice isoforms, we tested physiological predictions based on exon-reporter ratios. We proposed that exon-reporter ratios reflect VGCC peptide ratios and made predictions regarding presynaptic physiology based on biophysical and physiological data associated with the inclusion/exclusion of either exon 10 or 11 and exon 5 or 6 (Bell et al., 2025) and exon 34 (Chang et al., 2014; Lembke et al., 2017, 2019). We then tested these predictions against our own terminal-specific physiological data, both published (Chouhan et al., 2010, 2012; Lu et al., 2016; Justs et al., 2022) and unpublished (Table 2).

**Table 2.** Physiological data specific to each of the six motor neuron terminals

Terminal identity 1	Volume (μm^3^)	AZs/vol. (μm^−3^)	Total AZs (N_AZ_)	Q.C. (quanta)	AZ prob. (P_AZ_)	Ca^2+^/AP (10^6^ ions)	Ca^2+^/AP/AZ (10^3^ ions)	Firing (Hz)
	Justs 2	Present 3	Present 4	Present 5	Present 6	Justs 7	Present 8	Chouhan 9
MN6/7-Ib	311 ± 20	2.62 ± 0.22	813 ± 86	81.9 ± 4.8	0.101 ± 0.012	3.79 ± 0.30	4.66 ± 0.62	21.3 ± 0.5
MN13-Ib	392 ± 49	2.21 ± 0.31	864 ± 163	38.7 ± 9.5	0.045 ± 0.014	2.85 ± 0.33	3.30 ± 0.73	42.0 ± 1.1
MN12-Ib	366 ± 38	2.21 ± 0.56	808 ± 222	25.5 ± 2.5	0.032 ± 0.009	4.03 ± 0.33	4.99 ± 1.43	32.4 ± 1.7
MNSNb/d-Is, M6	90 ± 20	2.69 ± 0.36	242 ± 63	74.2 ± 3.8	0.306 ± 0.081	1.67 ± 0.29	6.89 ± 2.15	7.8 ± 0.6
MNSNb/d-Is, M13	97 ± 16	6.03 ± 1.78	584 ± 197	43.2 ± 5.6	0.074 ± 0.027	1.54 ± 0.23	2.64 ± 0.98	-
MNSNb/d-Is, M12	128 ± 29	4.13 ± 0.71	530 ± 151	62.3 ± 5.6	0.117 ± 0.035	2.43 ± 0.36	4.59 ± 1.47	7.8 ± 0.1
Is average					0.166 ± 0.071		4.71 ± 1.23	7.8 ± 0.3

These data represent new data or analyses, annotated as “present,” or a compilation from previous studies published by our laboratory annotated as “Justs” (Justs et al., 2022) or “Chouhan” (Chouhan et al., 2010, 2012). Whole terminal volume data (column 2; μm^3^) were generated through immunohistochemistry and confocal microscopy, as described previously (Lu et al., 2016; Justs et al., 2022) and published in Justs et al. (2022). AZ number per unit volume (μm^−3^) data (Column 3) were generated through reference to transmission electron microscopy data described previously (Lu et al., 2016; Justs et al., 2022) but mostly unpublished. The total AZ number for specific terminals (N_AZ_; Column 4) is the mathematical product of Columns 2 and 3. QC data were generated from terminal-specific TEVC recordings of EJCs and muscle-specific TEVC recordings of mEJCs, all in the presence of 2 mM CaCl_2_ added to HL6 (see Materials and Methods). The average probability of release from an individual AZ (P_AZ_; Column 6) was calculated by dividing QC by the total number of AZs. The number of Ca^2+^ ions entering each terminal (Column 7), and the method of calculation was reported previously (Justs et al., 2022). The average number of Ca^2+^ ions entering through each AZ in response to an AP (Column 8) is calculated by dividing total Ca^2+^ entry by the number of AZs. The endogenous firing frequency of each MN terminal (Column 9) was reported previously (Chouhan et al., 2012), calculated through the use of current-clamp electrodes recording simultaneously in adjacent muscles fibers in the presence of 2 mM CaCl_2_ added to HL6. Standard error of the mean (SEM) calculated according to propagation of uncertainty theory (Farrance and Frenkel, 2012) in Columns 4, 6, and 8.

**Figure 6.**
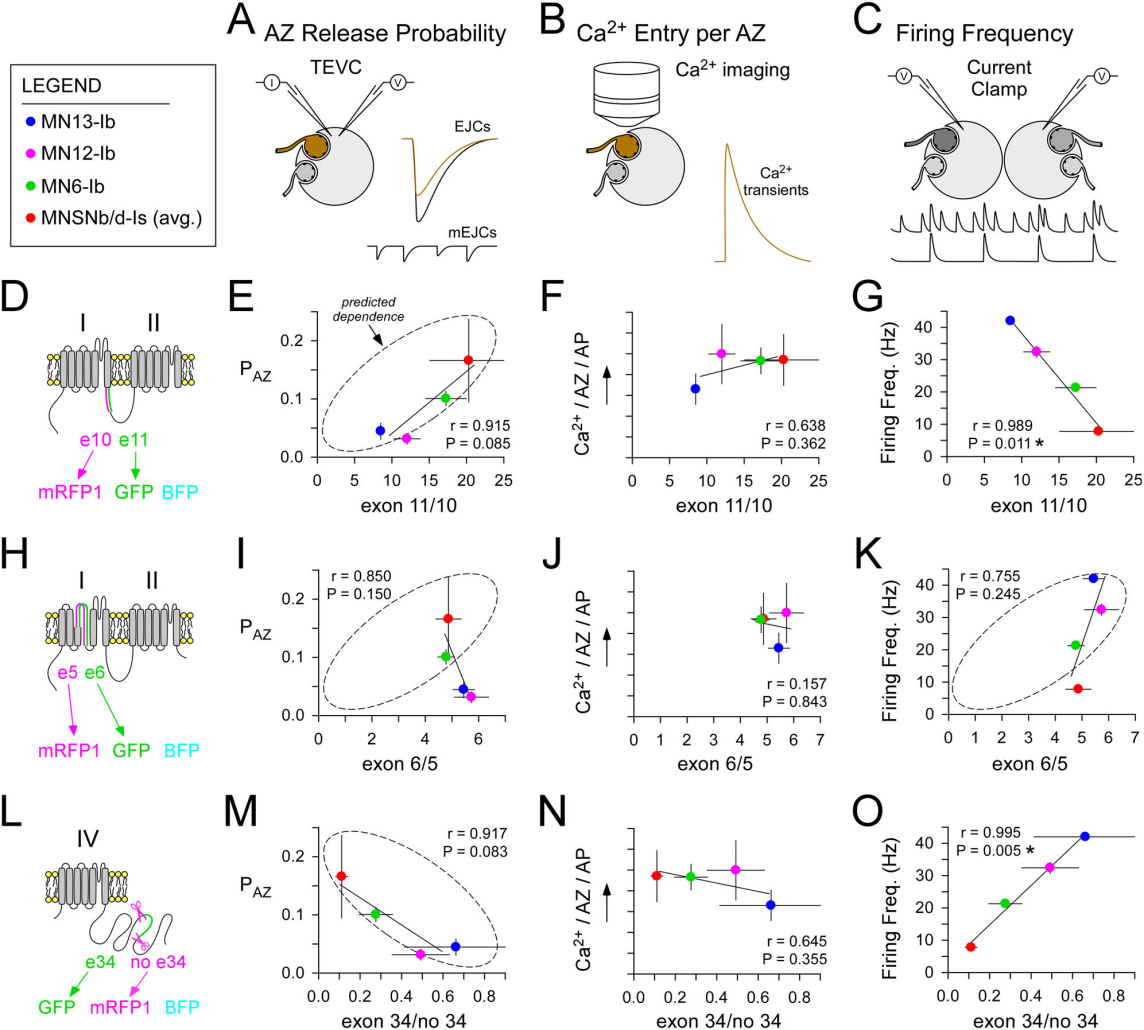
Physiological data validate several predictions from the reported exon biases. ***A–C***, Stylized diagrams representing the techniques used to collect physiological data plotted in the corresponding columns below each technique. Terminal-specific data in Table 2. ***A***, TEVC used to estimate a terminal's QC. The average probability of release at individual AZs (P_AZ_) is calculated as the QC for a single isolated AP divided by the average number of AZs (Table 2, column 6). ***B***, Ca^2+^ imaging with chemical Ca^2+^ indicators used to estimate the total number of calcium ions (Ca^2+^) that enter a terminal. The average number of Ca^2+^ ions entering each AZ during an AP (Ca^2+^_AZ_) is calculated as the total Ca^2+^ entry divided by the average number of AZs (Table 2, column 8). These values are considered overestimates due to Ca^2+^ influx through DmCa1D, and so Ca^2+^_AZ_ values are only represented in proportion on the ordinate. ***C***, Current-clamp recordings from adjacent muscle fibers used to estimate terminal-specific endogenous firing frequencies (Table 2, column 9). ***D***, ***H***, ***L***, Polypeptide context for the data in each adjacent row. ***E***, ***F***, ***G***, Physiological data (legend in ***O***) plotted against the exon 11 versus 10 ratio. Where a prediction has been made regarding the influence of the exon ratio, an ellipse has been drawn with the long axis denoting whether a negative or positive dependence is predicted. ***I***, ***J***, ***K***, Physiological data plotted against the exon 6 versus 5 ratio. ***M***, ***N***, ***O***, Physiological data plotted against the exon 34 present to 34 absent ratio. Means are plotted, with standard deviation error bars. Pearson's product–moment correlation coefficient was calculated to test the strength and direction of associations. Least-squares linear fits. Legend for terminal identity is shown at top left. Red, MNSNb/d-Is (average from three muscle fibers); green, MN6-Ib; pink, MN12-Ib; blue, MN13-Ib.

As previously described (Lu et al., 2016), we have used electron micrographs from multiple sets of serial sections through each terminal type to make estimates of the average number of AZs at each MN terminal (Table 2). These data were coupled with our estimates of QC and previous Ca^2+^ entry data (Justs et al., 2022), to make estimates of the “average” probability of release at each AZ (P_AZ_; Fig. 6*E*,*I*,*M*) and “average” Ca^2+^ entry at each AZ (Fig. 6*F*,*J*,*N*) across all terminal types (Table 2). No suggestion is made that release probability is uniform across AZs in the same presynaptic terminal, as nonuniformity is well documented at the *Drosophila* NMJ (Newman et al., 2022), but rather, P_AZ_ is terminal specific and a useful metric to compare with the proposed terminal-specific peptide ratios. Estimates of Ca^2+^ entry through Cac channels will be overestimates, as they are based on volume-averaged Ca^2+^ transients that are a product of Ca^2+^ entry through both Cac and DmCa1D, the Ca_v_1 homolog in *Drosophila* (Zheng et al., 1995; Krick et al., 2021). Estimates of endogenous firing frequency particular to each terminal were taken from Chouhan and others (Chouhan et al., 2010, 2012; Fig. 6*G*,*K*,*O*; Table 2).

Bell and others established that alternative splicing of exon 10 versus 11 affects Cac number and release probability (Bell et al., 2025). Specifically, excision of exon 11 (I–IIB), and not exon 10 (I–IIA), results in a reduction in neurotransmitter release and less Cac at each AZ. We would therefore expect a positive correlation between the ratio of exon 11 relative to exon 10 and P_AZ_, and this is what we observed (*r* = 0.915; *p* = 0.085; Fig. 6*E*) and is generally consistent with our estimates of Ca^2+^ entry at each AZ across terminals (*r* = 0.638; *p* = 0.362; Fig. 6*F*). While no predictions are made regarding firing frequency (but a prediction is made with regard to exon 5/6), we do see a significant negative association between firing frequency and the ratio of exon 11 to 10 (*r* = 0.989; *p* = 0.011; Fig. 6*G*).

Bell and others established that exon 6 (IS4B) is required for Cac localization to the AZ and tuning Cac's biophysical properties and that exclusion of exon 6 from the *cac* locus (but not exon 5; IS4A) is embryonic lethal (Bell et al., 2025). Consistently, exon-reporters show exon 6 to be present at high levels in all terminals. Of all the exons examined, the ratio of exon 6 to exon 5 showed the least variation, thus providing limited leverage for testing predictions. Based on the data of Bell and others, we might expect to see higher P_AZ_ with a higher level of exon 6, but this is not observed (Fig. 6*I*). Bell and others found that the presence of exon 6 is required to prevent inactivation during sustained depolarization, a property shown to be compatible with maintaining VGCC availability at high firing rates (Naranjo et al., 2015). Consistent with maintaining a pool of VGCCs available for activation in terminals, we found that terminals with a higher firing frequency tend to have a higher proportion of exon 6 (Fig. 6*K*), but this does not rise to the level of significance (*r* = 0.755; *p* = 0.245).

Lembke and others found no deficit in evoked release at the NMJ when exon 34 was excised from the endogenous locus, indicating that the sole Cac-RM isoform is competent to traffic to the plasma membrane and mediate AP-triggered neurotransmitter release (Lembke et al., 2017, 2019). Furthermore, under these conditions they reported Cac levels (the protein) to be diminished to 20% of wild-type levels, despite mRNA levels being undiminished. The implication is that Cac, in the “absence” of exon 34, is a potent facilitator of neurotransmitter release. Therefore, we predict that higher levels of exon 34 would result in “less” neurotransmitter release, and this is what we observe with a negative correlation between P_AZ_ and the reported levels of exon 34 (*r* = 0.917; *p* = 0.083; Fig. 6*M*). The published data cannot easily be extrapolated to make predictions regarding either Ca^2+^ entry or endogenous firing frequencies (Fig. 6*N*,*O*). In conclusion, where we find a substantial spread in exon-reporter ratios between neurons (11 vs 10 and 34 vs no-34) we also find substantial spread in terminal-specific physiological properties that might be predicted if exon-reporter ratios reflect peptide ratios.

## Discussion

Here, we investigated alternative splicing in *cac* across multiple neurons in vivo using fluorescent bichromatic exon-reporters. These reporters indicated a wide range of exon bias between neurons in cell-type–specific patterns, consistent from one animal to the next, suggesting that each neuron splices a unique and consistent portfolio of VGCC isoforms. Furthermore, we observed subcellular differences, potentially conveying information that might only otherwise be gleaned using isoform-specific antibodies or genetic tags (should they be available). However, these reporters are only valuable if it can be established that they do indeed report splicing biases. Serendipitously, each exon has previously been investigated for its physiological relevance in *Drosophila*, allowing us to make testable predictions for a set of MNs innervating body-wall muscles. These predictions were generally consistent with our Ca^2+^ entry data, release site probability data, and endogenous firing frequency data, indicating that these exon-reporters have the potential to reveal *cac* exon splicing biases, if not peptide biases.

Our exon-reporters indicate that exon 11 (I–IIB) is spliced in preference to exon 10 (I–IIA) in MN terminals and that exon 6 (IS4B) is spliced in preference to exon 5 (IS4A), findings consistent with the recent study of Bell et al. (2025). Where our data diverge from the 2025 study is in the magnitude of the bias of these mutually exclusive exons. For example, we interpret our data as indicating that for every 100 Cac VGCCs in Type Ib terminals, 95 contain exon 11 (95%) and only 5 contain exon 10 (5%). However, Bell and others indicated that only 67% of Cac would contain exon 11 while 33% would contain exon 10, i.e., exon 11 and 10 are present at a ratio of 2:1. Furthermore, we assessed exon 6 to be present in 83% of Cac in Type Ib terminals, with exon 5 present in the remaining 17%, yet Bell and others detected no sign of exon 5, i.e., 0% compared with our assessment of 17%. It is known that VGCCs with different pore-forming α_1_ subunits distribute to different subcompartments of a neurons (Alves et al., 2019), and splice isoforms may do the same (Maximov and Bezprozvanny, 2002), and so these exon-reporters may not capture subcompartments if they only provide a readout that is an average for the entire neuron. The capability of these reporters to provide subcellular information is discussed further below, but we might also consider the possibility that some Cac VGCC isoforms are missed when Cac quantification focusses on AZs. For example, the Cac splice isoform that resides in the lysosomal membranes (Tian et al., 2015) has yet to be identified. In a similar vein, if the acute presynaptic homeostatic plasticity (PHP) response draws on an intracellular “reserve pool” of Cac that is trafficked to AZs (Goel et al., 2019), then any isoform preferentially mobilized from these pools would be undercounted at AZs. As exon 11 (I–IIB) is necessary for the acute PHP response (Bell et al., 2025), then Cac isoforms containing exon 11 might be undercounted at AZs under resting conditions.

The low but nontrivial signal measured in the muscle using the reporter construct for exon 10 versus 11 is consistent with the low level of *cac* mRNA detected in third instar larval muscles fibers #1 and 4 [∼0.8% of neuronal total transcripts per million (TPM); Jetti et al., 2023]. The same exon-reporter expressed in perineural glia cells gave a strong signal indicative of exon 10 being present at ∼75% of Cac VGCCs, with exon 11 in the remaining 25% (Fig. 2*F*–*H*). While scRNA-seq has confirmed the presence of *cac* in ensheathing glia (scRNA-seq database Scope; scope.aertslab.org), a glial subtype encompassed by the *repo* pan-glial expression pattern (Doherty et al., 2009), *cac* isoform data are not available for glia. It is not known if Cac forms functional channels that traffic to the plasma membrane in either muscle or glia, but the prevalence of exon 10 in glial Cac VGCCs indicates that its function does not rely on GPCR signaling as exon 10 lacks the predicted Gβγ binding site (Fig. 1*D*).

While *cac* is homologous to α_1_ subunit genes within the vertebrate Ca_v_2 VGCC family, the exons studied here are without homology in vertebrate Ca_v_2 α_1_ subunits, yet they appear to serve a crucial role in providing functional diversity in *Drosophila*. In vertebrates, different Ca_v_2 α_1_ subunits contribute differently to the strength and plasticity of neurotransmitter release (Regehr and Mintz, 1994; Catterall and Few, 2008) as do the different splice isoforms of Ca_v_2 α_1_ subunits (Bourinet et al., 1999; Cingolani et al., 2023). In *Drosophila*, alternative splicing of *cac*'s many exons allows for functional specialization across different neural circuits, compensating for the limited number of Ca_v_2 α_1_ subunit genes. Thus, despite the lack of direct homologs, the study of these exons in *Drosophila* can offer valuable insights into how alternative splicing can generate a diverse range of VGCC isoforms and functions, with potential implications for understanding similar processes in more complex vertebrate systems.

A simplistic model of the mode of action of exon-reporter constructs is that cells fill with a mixture of fluorophores reflecting the composition of the pool of mRNA immediately outside the nucleus and that this reflects spliceosome bias. However, this is an unrealistic model as transcription rates change along with spliceosome bias throughout the lifetime of a neuron (Furlanis and Scheiffele, 2018). Such changes will evade detection by these reporter constructs as their signal is “integrative” in nature, providing a long-lasting trace of spliceosome activity. The constructs might be engineered to show greater temporal resolution through the inclusion of degradation signals, a strategy that would trade signal strength for temporal responsiveness. Alternatively, construct transcription might be conditionally activated using techniques such as GeneSwitch or Gal80^ts^ (Osterwalder et al., 2001; McGuire et al., 2003). A more acute approach might be to photobleach or photoconvert the fluorophores, allowing for quantification of newly translated fluorophore.

Our observation of differences in the exon-reporter ratio between soma and axon terminals (Fig. 5*F*,*I*,*L*) represents an intriguing phenomenon but requires careful consideration of the limitations of a reporter design that relies on translation of mRNA to signal spliceosome bias. As a large transmembrane protein, Cac must cotranslationally insert into the endoplasmic reticulum membrane before further processing, transport, and exocytosis before arriving in the presynaptic membrane. This contrasts with cytosolic proteins, such as the fluorophores generated off exon-reporter transcripts, which likely disperse passively throughout the cell. Passive diffusion seems most likely as the P2A motif between exon-derived peptides and fluorophores ensures that the fluorophores travel separately from the exon peptide, i.e., dissociated from any trafficking motifs. Isoform specificity in transcript transport coupled with local translation might give rise to subcellular differences in exon-reporter fluorophores, thus representing subcellular differences in cytosolic proteins, but we have been unable to propose a mechanism whereby the fluorophore gradients we observe should reflect subcellular differences in a large transmembrane protein such as Cac.

While we made predictions regarding average Ca^2+^ entry at individual AZs based on published neurotransmission data (Lembke et al., 2019; Bell et al., 2025), we could not make predictions regarding average VGCC number at individual AZs. Such an extrapolation is not sustainable as neither average Ca^2+^ entry per AZ, nor the average probability of release from an AZ (P_AZ_), is positively correlated with average VGCC number between AZs at different *Drosophila* nerve terminals (Medeiros et al., 2024). For example, while Ca^2+^ entry at the AZs of MNSNb/d-Is terminals is greater than that at the AZs of MN6-Ib (Lu et al., 2016; He et al., 2023) and the probability of release is between two- and threefold higher at the AZs of MNSNb/d-Is (Lu et al., 2016; Newman et al., 2022), the VGCC number is no different (He et al., 2023; Medeiros et al., 2024). In other words, factors apart from VGCC number play a major role in determining Ca^2+^ entry at different terminals, e.g., differences in VGCC splice isoforms, accessory subunits, or posttranslational modifications. Therefore, without identifying terminals with different numbers of Cac VGCCs in their AZs, our information on exon bias between terminals cannot be leveraged to determine which exons might influence the Cac VGCC number.

Exon 34 is excluded from only 1 of the 18 annotated Cac isoforms (Cac-RM). Consistently, Jetti et al. (2023) reported low levels of Cac-RM mRNA from patch-seq RNA profiling of material extracted from the somata of identified MNs in third instar larva. They reported the Cac-RM isoform to be present at only 12.9% of TPM in a Type Ib representative (MN1-Ib) and only 11.4% in a Type Is representative (MNISN-Is). In contrast, our data, also from the third instar larva, indicate that Cac-RM is present at 60–90%. The data of Lembke et al. (2019) lend plausibility to the notion that Cac-RM might be present at high proportions as they demonstrated *Drosophila* are viable when exon 34 is deleted, and Cac-RM is the only isoform available, i.e., present at 100%. However, the other 17 isoforms are by no means redundant, as exon 34 deletion results in a disrupted motor pattern output and slower locomotion in larvae. Surprisingly, despite measuring an 80% reduction in Cac protein in the adult nervous system when exon 34 was deleted, Lembke and others did not find a deficit in evoked neurotransmitter release at the larval NMJ.

Our exon-reporters indicated that MNSNb/d-Is terminals, with the lowest proportion of *cac* isoforms with exon 34, were often missing from muscle fibers #6, 7, 13, and 12. Missing terminals may represent a developmental phenotype arising from the presence of competitive peptides; either in-frame or out-of-frame. Out-of-frame fluorophore peptides are common to all of these reporters, and so it is unlikely that they are responsible for the phenotype. Along with high levels of mRFP1, MNSNb/d-Is will contain high levels of in-frame exons 33 and 35 peptides (Fig. 4*H*) implicating roles for either peptide or both in the “missing terminals” phenotype. Two of the three peptides considered here are distinguished as binding partners in known signaling pathways. First, exon 34 is bound by *tdph*, the *Drosophila* ortholog of transcription factor TAR RNA-binding protein (TDP-43), and there are phenotypic similarities between *tdph* mutants and exon 34(7) excision mutants (Hazelett et al., 2012; Chang et al., 2014; Lembke et al., 2017, 2019). However, the role of exon 34 in transcriptional regulation is far from clear. Furthermore, MNSNb/d-Is, with its reported low level of exon 34, would be expected to have the least amount of exogenous exon 34 (with 3′ end translated out-of-frame; Fig. 4*H*), yet it is the terminals of MNSNb/d-Is that go missing. The second distinguished peptide, coded by exon 35, contains a binding domain for adaptor protein complex-1 (AP-1; Lembke et al., 2019). AP-1 binds the same domain in the carboxy terminus of mouse Ca_v_2.2 and assists its trafficking to the plasma membrane (Macabuag and Dolphin, 2015), raising the possibility that competitive binding with an exon 35 peptide disrupts Cac trafficking to the plasma membrane in this study.

Our observation of differential exon biases between MB lobes (Fig. 3*B*–*E*) suggests differential involvement of VGCC isoforms in olfactory information processing. KCs are intrinsic to the MB where they fasciculate as they course through the peduncles and terminate in separate branches of the dorsal and medial lobes. KCs that terminate in the γ lobe are associated with short-term memory, while KCs of the α′ and β′ lobes play a role in memory consolidation and KCs of the α and β lobes are crucial for long-term memory (Davis, 2023). The preferential expression of exon 10 in the γ lobe and α′ and β′ lobes suggests that exon 10 (I–IIA) may play specific roles in both short-term memory and memory consolidation. Conversely, the preferential expression of exon 11 in α and β lobes suggests that exon 11 (I–IIB) may be necessary for the long-term synaptic changes required for memory storage. Therefore, a fine-grained map of exon bias across lobes of the MB might offer substantial insight into the contribution of VGCC α_1_ subunit splice isoform diversity to olfactory information processing.

## Synthesis

Reviewing Editor: Katrina Choe, McMaster University Decisions are customarily a result of the Reviewing Editor and the peer reviewers coming together and discussing their recommendations until a consensus is reached. When revisions are invited, a fact-based synthesis statement explaining their decision and outlining what is needed to prepare a revision will be listed below. The following reviewer(s) agreed to reveal their identity: NONE. Note: If this manuscript was transferred from JNeurosci and a decision was made to accept the manuscript without peer review, a brief statement to this effect will instead be what is listed below.

This manuscript describes a novel technique that enables cellular level identification of distinct splicing variants of Cacophony, a type of calcium channels expressed in drosophila, using fluorescent proteins that correspond to different splice variants.

Reviewers found the method a potentially useful technique to study cell type-specific splice isoform expression, which could be of interest to other laboratories. Both reviewers found the design of the splicing reporter smart and clever, and the experimental results showing cellular level expression patterns to be credible. However, the reviewers also pointed out some shortcomings of the study. Specifically, both reviewers were less convinced of its application for cell compartment specific localization and assessing physiological functions in a circuit context. Furthermore, reviewer 2 had concerns with validation and experimental design of specific datasets, and noted a lack of functional data on the different isoforms of Cacophony.

Please see below for specific comments from each reviewer:

Reviewer 1

Major concerns

(1) A comparison of expression levels in subneuronal compartments was attempted to study expression bias in a cell compartment specific manner, but it remains unclear why the GAL4 mediated overexpression of splicing reporters should phenocopy the sub-neuronal localization of endogenous channel isoforms. Also, some of the findings on compartment-specific isoform localization contradict published work, e.g. exon 5 is not found at presynaptic terminals onto muscle 6, but the reporter provides positive expression results. This indicates clear limitation of the method but is not even discussed. Please add a critical evaluation of the limitations of the technique.

(2) The finding that MNs to different muscles express different ratios of the exon variants 10 and 11 is not fully supported by the data. Expressing two splicing reporters under the control of GAL4 in the same neurons can have many effects on the expression levels of either one. It is credible that the ratios of exon 11/10 are always well above 5, meaning exon 11 seems to be spliced far more frequently in motor neurons, but to use such indirect measures to further distinguish motor neuron types seems error prone. This point seems particularly critical, because figure 7 states that the ratio of exon 11/10 is proportional to release probability with Is boutons showing a significantly higher release probability than 1b boutons. However, in figures 2H and 4H the exon 11/10 ratios are identical for 1s and 1b boutons on muscle 6. Given that it is well known that muscle 6 1s boutons have a higher release probability than muscle 6 1b boutons figures 2h and 4h show that the reported exon 11/10 rations obtained by this technique do not correlate with release probability. Similarly, according to table 2, the 1s boutons on muscles 6, 12, and 13 show vastly different release probabilities, but all show similar exon 11/10 ratios. Thus, the correlation between Pr and 11/10 ratios does also not apply here. Therefore, it remains unclear how the data shown in different figures panels can be reconciled. Please either down tone this results substantially or remove it.

(3) The power of the method is heavily overstated. For example, the reporter ratios for exon 11/10 at neuromuscular boutons are between 17 and 30, but the reported difference of channels in active zones with exon 11 versus channels with exon 10 is twofold (Bell et al., 2024). Moreover, the reporter-based differences between exons 5 and 6 are around 5-fold, but Bell et al. (2024) have demonstrated that exon 5 is entirely missing from motor neuron terminals. Moreover, the introduction states on lines 95-97 that this technique allows for probing neuron-specific functions of previously undistinguished protein motifs in a VGCCs. First, the method does not allow to test for function, but instead, it can give a first hint on cell type specific expression. Second, the protein motifs investigated here have previously been analyzed. Such overstatements are repeated throughout. Another example is line 320 in the discussion '. ...providing insight into the role of alternative peptides in polypeptide function and circuit context'. No, there is no direct functional insight from this technique. Please remove overstatements of method utility from the text.

(4) the subcellular bias in reporter expression levels should be evaluated with great care. Given that there is no satisfactory mechanistic explanation for this, it should not be used to draw conclusions on the sub-cellular localization of different splice isoforms. It can be reported as a yet not understood phenomenon.

(5) The long discussion on mRNA translation in the axon terminal and differential mRNA transport for the different reporters is not evidence based, but sole speculation. Potential other mechanisms that can explain differential reporter localization are not considered in depth. Furthermore, although mRNA in the axon terminal has been reported before, there are no published evidence that this happens for large transmembrane proteins, and given the genesis, maturation, and transport of VGCC proteins, it seems highly unlikely. Without further experimental evidence, this part of the discussion must be substantially shortened, down-toned, and alternative explanations should be listed.

(6) The section stating that in vivo properties correlate with the different ratios of cacophony splice isoforms is intruiging but requires major revision. First, it is physiologically absolute unclear, how the axon terminal exon ratios should affect motor neuron firing frequency. Motor neuron firing frequency can be affected be somatodendritic VGGCs as reported for larval Drosophila MNs by Worrell and Levine (2008), or it can be fine-tuned by axonal VGCCs (Kadas et al., 2017), but axon terminal VGCC isoforms do not regulate motor neuron firing rates. Please remove these analyses from the ms. Second, as mentioned above, there are contradictions between figures 2 and 4 on one side and figure 7. Please clarify. Also, it is not explained how release probability was determined in this study. Third, the calcium imaging data as presented require revision. The method to calculate the number of calcium ions entering the terminal is error prone and not necessary. Even ratiomentric measurements cannot give true calcium concentrations without many additional controls. Why not report delta F/ F? Deriving active zone calcium by correcting total bouton calcium for the number of active zones that are counted by EM from other animals is error prone, too. Here, cacophony isoform function is diluted by calcium influx through other VGCCs. All of these complications can easily be avoided by using genetic constructs that report calcium entry into active zones (readily available and used by others) and report delta F / F in active zones. This would eliminate major sources for error and the complication of action potential driven calcium influx through Dmca1D. If the authors want to stick to their approach, the least would be to normalize the y-axis to maximum and not report number of calcium ions per active zone.

Minor comments

(1) If possible at all, it would be great to improve the quality of the images of immunohistochemical label in Fig. 1. The main points come across, i.e. exon expression bias likely exists between cacophony isoforms containing exon 10 versus 11 in the optic lobes versus central brain regions including the mushroom bodies, but presentation clarity (selective enlargements, single sections etc.) and image quality could be improved.

(2) Image quality for motor neurons (MNs) in Fig. 2 is much better. The results that in MNs cacophony variants containing exon 11 are favored over exon 10, but in glia the opposite expression bias is observed is striking. However, it would be important to report right here that the exchange of fluorophores does not alter the result. This very important result was reported later in the manuscript (Fig. 4). I suggest to move the results shown in figure 4 to figure 2 as this lends credibility to the results. This way it would be clearer to the reader that the results actually match.

(3) Muscle expression: as far as I am aware, crawling muscles do not contain functional cacophony channels. How does the weak fluorescence that reports cacophony variant expression bias in muscle fit? Is there always baseline splicing of anything expressed artificially? This must be discussed very carefully.

(4) Can the authors speculate about functional underpinnings of cacexon10 versus cacexon11 expression in MNs versus glia? At least some discussion on this finding would be great.

(5) Figure 3: To me it appears more sensible to label the mushrooms body lobes in the single color images rather than in the overlay. This would make it easier for the reader to relate the label to the respective structure. Is there really no fluorescence that hints at expression of the respective other exon in the mushroom body lobes? It is important to show this in the images of the figure and not only report that there was no other label that hints at expression of cacophony splice isoforms with the respective other exon. And if there was label, even faint, please state this. As different biophysical properties can likely be attributed to the different channels depending on exon incorporation, it appears important to report this.

(6) Fig. 5: expression of cac-RM (1 out of 18 variants) appears to be favored over all other cacophony variants (17 out of 18 variants). Please show that the respective terminals were often missing. The top figure panel in Fig. 5I shows a process that is labeled with an asterisk. However, neither red nor green fluorescence is shown in this process indicating that cacophony is not expressed at all. Therefore, this process does not qualify to make this point. Please show other muscles that substantiate the finding of terminal loss.

(7) Why would a non-inactivating Cav2 channel favor firing at higher rates? What subneuronal compartment are we talking about? Action potentials are generated and propagated in the axon. What does this have to do with the synapse? Or do the authors mean that MN burst firing during crawling needs a non-inactivation cacophony channel at the presynapse for faithful synaptic transmission during the entire firing burst? I do not understand this parameter.

(8) Discussion, p. 12, line 389: That action potential width may have an effect on the amount of transmitter that is released is undisputed, but does this have to do with cacophony? I completely agree that cacophony can have an impact on AP width, but only if cacophony channels participate in AP shaping. For this they would have to be expressed in the axon. Are there data that point in that direction? This should be explained if AP width is used as parameters.

(9) I encourage discussion of the functional implication of the actual differences of the alternatively expressed cacophony exons. For example, according to Smith et al. 1998, cacophony isoforms containing exon 11 versus exon 10 possess a binding site for G-protein βγ subunits. Similarly, the present study reports a difference in the number of positively charged amino acids depending on whether exon 5 or 6 is spliced in. Towards the end of the discussion, olfactory processing and memory formation are discussed without clear hypotheses. Wouldn't it be worthwhile to rather include the known differences in the discussion?

Reviewer 2

Specific points

1. The authors state that these "transgenic bichromatic exon-reporters are composed of DNA that mimics a limited stretch of a gene's open reading frame (ORF) along with DNA sequences encoding two distinct fluorophores". I wonder if using a "limited stretch of a gene's ORF" recapitulates the trafficking and expression patterns of Cacophony isoforms. The authors refer to this approach being previously used (lines 85 and 86). Did any of these studies provide validation on whether the approach mimicked the expression patterns of the isoforms being examined?

2. Why were type-II and type-III boutons excluded? Did the authors not look at these boutons because n-syb-GAL4 does not express in them or was there another reason? Is Cacophony expressed in those types of boutons? It would be interesting to know if different Cacophony isoforms were more likely to be expressed in terminals that release octopamine (type-II) or insulin (type-III) in comparison to those that release glutamate (type-Ib and type-Is).

3. The study lacks functional data on the importance of the different Cacophony isoforms. Figure 7 does provide some correlations of electrophysiological data and calcium imaging data from presynaptic terminals that have different ratios of isoforms expressed, but it is hard to make conclusions about function without loss-of-function data. The authors do refer to a recent eLife paper (Bell et al., 2024), which does allow for some conclusions to be made.
