# Supplementary material for: Bichromatic Exon-Reporters Reveal Voltage-Gated Ca2+-Channel Splice–Isoform Diversity across Drosophila Neurons In Vivo
Source: eNeuro. 2025 Aug 14;12(8):ENEURO.0582-24.2025. doi: 10.1523/ENEURO.0582-24.2025 (PMC12370356; doi:10.1523/ENEURO.0582-24.2025)
Supplement: Figure 1-1 — Exon reporter construct nucleotide sequences. The nucleotide sequence of each exon reporter has been annotated to allow substantiation of the intended effect of adding nucleotides to change frame. A. cac_10_11_GFP_TagRFP-TB. cac_10_11_GFP_mRFP1C. cac_10_11_mRFP1_GFPD. cac_5_6_GFP_mRFP1E. cac_34_GFP_mRFP1F. cac_34_GFP_mRFP1_remade Download Figure 1-1, DOCX file. [file eneuro-12-ENEURO.0582-24.2025-s002.docx]

***Construct Sequences***

**A. cac_10_11_GFP_TagRFP-T**

**GCGGCCGC**caaa**ATGtg**GACCAACGACGCATTAGGTTCAGCATTTAATTGGATATATTTCGTGCCTCTTATAGTTATAGGCTCATTTTTTATGCTCAACTTAGTTCTTGGTGTCCTTAGTGGGTAAGTTGATCGCGAATTGCTTTATTTTAACGATGAAGCCACTTCTACACCAGTTCACATTATGTTCTACTTATAATTTCGATTACTTCCGTTGAGAAAGAGAGTAAAAAAAACAAAAAACAAAAAAAAAAAAAAATATGGAAAAATGGGATCTGAAAATACTGCATACATATGTTTAGATCATTTCTATATGGAATGTCGTCGTGTGTTTTTTATGACGCATTTAAATATTTTTGTAAAGATGAAATGTAAAGAAAGTGTGTCCAATTGCCAGATTATACATATGTAATATATCGGTTTTACGTAGAGCTACGTGCATGTGCATACACGGTGAAAAAAAAAATGATAAAAAATAAGTCAATTTGGCTGTAAATTATTCAAAATTTTCGCCTGTTGCCGGACGATTGAAATATATGTATGTACGTGTTTGATATACTGTCATTTTGATTTGCATTCAACTTGTACGTTTCGTTATATATTTGTTTTTATCTTTTTCCGAAATTTTGTTCAATGCATTTAACTATCGTATTCCCGAATTCA**GA**GAGTTTTCGAACGAACGAAATCGTGTCGAACGTCGCATGGAGTTTCAAAAATGCCGCTTTCGGGCCATGTTTCAGACAGCAATGGTCTCG**TA**CCTTcGACTGGATCACACAAGCAGGTTTATACAACTAGATCTATATCTAAGTGCAACCAAGTAAACTGCCATAATCAACCCGAACGTCATACAACACAACCTGCAATTATAACTAATTATAACTATTATCGGCCAAAAAAAAACAGAACAATAAGTCAACTCGTATCGTAATGCTAATGCTAATGCTATCGAATCCGAGCGAGTTCATTGAGCTAAACACCCATCTAATTAAACCAAAAAAAAAAACAATTCCGACCTATACTTCCTTAACCAGTTTATAAAGAACGTGTTTGATTTACTGCTAGTTATGTATTCTTAAATAGATCGCAAGCAATTCCCCGGCTACAAAGAGTTCTGGTACCCAAAACTAACTTAACTATCAATTACAATACAACTACTATGTACTTCCAATGAACCGAATTAATAATATTAACTGCGAAATGCCGTTCACCCCACTATAGTTTCTAACTAACCAACCAACCAAACAATCAATAATCTAATCCATATATACTCTGCAGTGCGTAATGCATATTATATTCTCTGCATTTTGCCACACACTTAACCCGATTTTTACTACCATAGCGATAAAGTTGAATTCCCCCCCCCTCCCTGTCAAAATAAACCAAACTAACCAAATACATAGCTATTTCAACTGCAGTTAGCCAAATTAACCTCCAGTTATATACTTATACCCGCCAGCGGACTCGTGAATTGTTGTTGAGTCTCGGCCTCGTTCTCGATATCTTGGCTAATATAGTACACTCCCTATACACTATATACACTGATAGAACAGTGCACCCACCTATCAATATCAATGTTCTGTCCCCGGTTAACTATCGCCATCAAAATGTATCATCGCTCCAGACTCAGAGACTTCAAGACTCGCAGACTCCCAGCGAATCAGGGCAAAATTCAGAGTTATTCAAGACCAGATGACGCTATGGCATCATCACCCAATGCGTACTCTATATATTTATTTGTCGCCACCGAATAAATCATTTTATATATGCTTGTATGCACACATATAATCTAACACATTTCCCTTCTTTTATATATATTTTTCCTTGACTTTTGACCTCATTGAAGGTTCCCGTCGAGGAGGCGCAATAAATATATACATATATATACAATATATGTGTTAAATTGCATTTCTGTTACTTCATTTCCAGTAAATGATTTCGACTTTCTGTTTATGCCCGTTTGTTGTGTTCTAAAAAACAAAAAAAAAAACAAAGCACTGGAATATTCTTGATTTTACGAATCTTTAAGTTATTCTTCTCTGTTGATTACACTCGTACAAAAACAATGGCGAAAGTATGATGTGAACTTTGATATTGTAATACTGAATACCAAAGCATACATGATCGTTTTAGCAATCAATCAATCCTGTTTTTTTGGTTTATCACGTTCTATTTAATTATTTTTTGTA**GT**GAATTCGC**AAAAGAACGAGAAA**AAGTAGAAAATAGACAAGAGTTTCTTAAACTTAGAAGGCAGCAGCAACTAGAAAGAGAGTTAAACGGCTATGTTGAATGGATTTGTAAAG**CTG**GTATAGTATTTACCCCTATTTCTTGTTTGTAAATCTATATATACACACACACACAATGACTAAACTCATGAATAACTCTCATACACACATATATACAAACATAAAAATTTAAACACTATGCGCCCCCATATGTTCATCCACATCCATATGTACATATATATATAAATTATAAAAACGACATTTATATCAGGTCCTTTTCTATGACTATAAAACTGAATTCAATGAAAATAGGCCAACTATAATCACATAAAATATTTTTTTAAAAATTCGAACAGGGATCACAAAAAATGCCTAACAAAAGATTCTTCTTTAATGTCATTGCTTCACTGAAAGATACATTAACTAAATATCGTTTATATCATTTTCATATAAAATGACGATATATTCATGCATATTTAAAGTGATACAAGAATAAAATATACGTTGTATTGAGATGTTCCCTAATAGCCAGCACACTTTTGGCACATTCTTAGCATCCCTCTCCTCATAGAATTGAAACCCTCGAACCTATATACATTGCCAAATTTGAAAGTGAATAAATTCCTCAATATACTCAGAATTAAAATAGTTTTCGTGGAAATATGGACATATATCCCTCATGCCGCCCCCAATATATATACATCTACCCACATTGACTATAATGTACATACGAGTATATATCCTTTCTGCTTATGTGTTGATCAAATCGATTCCCTTATTGTGACCCATCACCAATCACTCGATTTTAATGCTATTTCAGAGGAGGTAATCCTGGCCGAGGAGCGCACCACGGAAGAAGAGAAAATGCACATAATGGAgGGAAGCGGAgccaccaacttctccctgctgaagcaggccggcgacgtggaggagaaccccggccccatggtgagcaagggcgaggagctgttcaccggggtggtgcccatcctggtcgagctggacggcgacgtaaacggccacaagttcagcgtgtccggcgagggcgagggcgatgccacctacggcaagctgaccctgaagttcatctgcaccaccggcaagctgcccgtgccctggcccaccctcgtgaccaccctgacctacggcgtgcagtgcttcagccgctaccccgaccacatgaagcagcacgacttcttcaagtccgccatgcccgaaggctacgtccaggagcgcaccatcttcttcaaggacgacggcaactacaagacccgcgccgaggtgaagttcgagggcgacaccctggtgaaccgcatcgagctgaagggcatcgacttcaaggaggacggcaacatcctggggcacaagctggagtacaactacaacagccacaacgtctatatcatggccgacaagcagaagaacggcatcaaggtgaacttcaagatccgccacaacatcgaggacggcagcgtgcagctcgccgaccactaccagcagaacacccccatcggcgacggccccgtgctgctgcccgacaaccactacctgagcacccagtccgccctgagcaaagaccccaacgagaagcgcgatcacatggtcctgctggagttcgtgaccgccgccgggatcactctcggcatggacgagctgtacaag**taaAA**GGAAGCGGAgccaccaacttctccctgctgaagcaggccggcgacgtggaggagaaccccggccccgtgtctaagggcgaagagctgattaaggagaacatgcacatgaagctgtacatggagggcaccgtgaacaaccaccacttcaagtgcacatccgagggcgaaggcaagccctacgagggcacccagaccatgagaatcaaggtggtcgagggcggccctctccccttcgccttcgacatcctggctaccagcttcatgtacggcagcagaaccttcatcaaccacacccagggcatccccgatttctttaagcagtccttccctgagggcttcacatgggagagagtcaccacatacgaagacgggggcgtgctgaccgctacccaggacaccagcctccaggacggctgcctcatctacaacgtcaagatcagaggggtgaacttcccatccaacggccctgtgatgcagaagaaaacactcggctgggaggccaacaccgagatgctgtaccccgctgacggcggcctggaaggcagaaccgacatggccctgaagctcgtgggcgggggccacctgatctgcaacttcaagaccacatacagatccaagaaacccgctaagaacctcaagatgcccggcgtctactatgtggaccacagactggaaagaatcaaggaggccgacaaagagacctacgtcgagcagcacgaggtggctgtggccagatactgcgacctccctagcaaactggggcacaaacttaatggcatggacgagctgtacaagtaa**ACCGGT**

**B. cac_10_11_GFP_mRFP1**

**GCGGCCGC**caaa**ATGtg**GACCAACGACGCATTAGGTTCAGCATTTAATTGGATATATTTCGTGCCTCTTATAGTTATAGGCTCATTTTTTATGCTCAACTTAGTTCTTGGTGTCCTTAGTGGGTAAGTTGATCGCGAATTGCTTTATTTTAACGATGAAGCCACTTCTACACCAGTTCACATTATGTTCTACTTATAATTTCGATTACTTCCGTTGAGAAAGAGAGTAAAAAAAACAAAAAACAAAAAAAAAAAAAAATATGGAAAAATGGGATCTGAAAATACTGCATACATATGTTTAGATCATTTCTATATGGAATGTCGTCGTGTGTTTTTTATGACGCATTTAAATATTTTTGTAAAGATGAAATGTAAAGAAAGTGTGTCCAATTGCCAGATTATACATATGTAATATATCGGTTTTACGTAGAGCTACGTGCATGTGCATACACGGTGAAAAAAAAAATGATAAAAAATAAGTCAATTTGGCTGTAAATTATTCAAAATTTTCGCCTGTTGCCGGACGATTGAAATATATGTATGTACGTGTTTGATATACTGTCATTTTGATTTGCATTCAACTTGTACGTTTCGTTATATATTTGTTTTTATCTTTTTCCGAAATTTTGTTCAATGCATTTAACTATCGTATTCCCGAATTCA**GA**GAGTTTTCGAACGAACGAAATCGTGTCGAACGTCGCATGGAGTTTCAAAAATGCCGCTTTCGGGCCATGTTTCAGACAGCAATGGTCTCG**TA**CCTTcGACTGGATCACACAAGCAGGTTTATACAACTAGATCTATATCTAAGTGCAACCAAGTAAACTGCCATAATCAACCCGAACGTCATACAACACAACCTGCAATTATAACTAATTATAACTATTATCGGCCAAAAAAAAACAGAACAATAAGTCAACTCGTATCGTAATGCTAATGCTAATGCTATCGAATCCGAGCGAGTTCATTGAGCTAAACACCCATCTAATTAAACCAAAAAAAAAAACAATTCCGACCTATACTTCCTTAACCAGTTTATAAAGAACGTGTTTGATTTACTGCTAGTTATGTATTCTTAAATAGATCGCAAGCAATTCCCCGGCTACAAAGAGTTCTGGTACCCAAAACTAACTTAACTATCAATTACAATACAACTACTATGTACTTCCAATGAACCGAATTAATAATATTAACTGCGAAATGCCGTTCACCCCACTATAGTTTCTAACTAACCAACCAACCAAACAATCAATAATCTAATCCATATATACTCTGCAGTGCGTAATGCATATTATATTCTCTGCATTTTGCCACACACTTAACCCGATTTTTACTACCATAGCGATAAAGTTGAATTCCCCCCCCCTCCCTGTCAAAATAAACCAAACTAACCAAATACATAGCTATTTCAACTGCAGTTAGCCAAATTAACCTCCAGTTATATACTTATACCCGCCAGCGGACTCGTGAATTGTTGTTGAGTCTCGGCCTCGTTCTCGATATCTTGGCTAATATAGTACACTCCCTATACACTATATACACTGATAGAACAGTGCACCCACCTATCAATATCAATGTTCTGTCCCCGGTTAACTATCGCCATCAAAATGTATCATCGCTCCAGACTCAGAGACTTCAAGACTCGCAGACTCCCAGCGAATCAGGGCAAAATTCAGAGTTATTCAAGACCAGATGACGCTATGGCATCATCACCCAATGCGTACTCTATATATTTATTTGTCGCCACCGAATAAATCATTTTATATATGCTTGTATGCACACATATAATCTAACACATTTCCCTTCTTTTATATATATTTTTCCTTGACTTTTGACCTCATTGAAGGTTCCCGTCGAGGAGGCGCAATAAATATATACATATATATACAATATATGTGTTAAATTGCATTTCTGTTACTTCATTTCCAGTAAATGATTTCGACTTTCTGTTTATGCCCGTTTGTTGTGTTCTAAAAAACAAAAAAAAAAACAAAGCACTGGAATATTCTTGATTTTACGAATCTTTAAGTTATTCTTCTCTGTTGATTACACTCGTACAAAAACAATGGCGAAAGTATGATGTGAACTTTGATATTGTAATACTGAATACCAAAGCATACATGATCGTTTTAGCAATCAATCAATCCTGTTTTTTTGGTTTATCACGTTCTATTTAATTATTTTTTGTA**GT**GAATTCGC**AAAAGAACGAGAAA**AAGTAGAAAATAGACAAGAGTTTCTTAAACTTAGAAGGCAGCAGCAACTAGAAAGAGAGTTAAACGGCTATGTTGAATGGATTTGTAAAG**CTG**GTATAGTATTTACCCCTATTTCTTGTTTGTAAATCTATATATACACACACACACAATGACTAAACTCATGAATAACTCTCATACACACATATATACAAACATAAAAATTTAAACACTATGCGCCCCCATATGTTCATCCACATCCATATGTACATATATATATAAATTATAAAAACGACATTTATATCAGGTCCTTTTCTATGACTATAAAACTGAATTCAATGAAAATAGGCCAACTATAATCACATAAAATATTTTTTTAAAAATTCGAACAGGGATCACAAAAAATGCCTAACAAAAGATTCTTCTTTAATGTCATTGCTTCACTGAAAGATACATTAACTAAATATCGTTTATATCATTTTCATATAAAATGACGATATATTCATGCATATTTAAAGTGATACAAGAATAAAATATACGTTGTATTGAGATGTTCCCTAATAGCCAGCACACTTTTGGCACATTCTTAGCATCCCTCTCCTCATAGAATTGAAACCCTCGAACCTATATACATTGCCAAATTTGAAAGTGAATAAATTCCTCAATATACTCAGAATTAAAATAGTTTTCGTGGAAATATGGACATATATCCCTCATGCCGCCCCCAATATATATACATCTACCCACATTGACTATAATGTACATACGAGTATATATCCTTTCTGCTTATGTGTTGATCAAATCGATTCCCTTATTGTGACCCATCACCAATCACTCGATTTTAATGCTATTTCAGAGGAGGTAATCCTGGCCGAGGAGCGCACCACGGAAGAAGAGAAAATGCACATAATGGAgGGAAGCGGAgccaccaacttctccctgctgaagcaggccggcgacgtggaggagaaccccggccccatggtgagcaagggcgaggagctgttcaccggggtggtgcccatcctggtcgagctggacggcgacgtaaacggccacaagttcagcgtgtccggcgagggcgagggcgatgccacctacggcaagctgaccctgaagttcatctgcaccaccggcaagctgcccgtgccctggcccaccctcgtgaccaccctgacctacggcgtgcagtgcttcagccgctaccccgaccacatgaagcagcacgacttcttcaagtccgccatgcccgaaggctacgtccaggagcgcaccatcttcttcaaggacgacggcaactacaagacccgcgccgaggtgaagttcgagggcgacaccctggtgaaccgcatcgagctgaagggcatcgacttcaaggaggacggcaacatcctggggcacaagctggagtacaactacaacagccacaacgtctatatcatggccgacaagcagaagaacggcatcaaggtgaacttcaagatccgccacaacatcgaggacggcagcgtgcagctcgccgaccactaccagcagaacacccccatcggcgacggccccgtgctgctgcccgacaaccactacctgagcacccagtccgccctgagcaaagaccccaacgagaagcgcgatcacatggtcctgctggagttcgtgaccgccgccgggatcactctcggcatggacgagctgtacaag**taaAA**GGAAGCGGAgccaccaacttctccctgctgaagcaggccggcgacgtggaggagaaccccggccccatggcctcctccgaggacgtcatcaaggagttcatgcgcttcaaggtgcgcatggagggctccgtgaacggccacgagttcgagatcgagggcgagggcgagggccgcccctacgagggcacccagaccgccaagctgaaggtgaccaagggcggccccctgcccttcgcctgggacatcctgtcccctcagttccagtacggctccaaggcctacgtgaagcaccccgccgacatccccgactacttgaagctgtccttccccgagggcttcaagtgggagcgcgtgatgaacttcgaggacggcggcgtggtgaccgtgacccaggactcctccctgcaggacggcgagttcatctacaaggtgaagctgcgcggcaccaacttcccctccgacggccccgtaatgcagaagaagaccatgggctgggaggcctccaccgagcggatgtaccccgaggacggcgccctgaagggcgagatcaagatgaggctgaagctgaaggacggcggccactacgacgccgaggtcaagaccacctacatggccaagaagcccgtgcagctgcccggcgcctacaagaccgacatcaagctggacatcacctcccacaacgaggactacaccatcgtggaacagtacgagcgcgccgagggccgccactccaccggcgcctaa**ACCGGT**

**C. cac_10_11_mRFP1_GFP**

**GCGGCCGC**caaa**ATGtg**GACCAACGACGCATTAGGTTCAGCATTTAATTGGATATATTTCGTGCCTCTTATAGTTATAGGCTCATTTTTTATGCTCAACTTAGTTCTTGGTGTCCTTAGTGGGTAAGTTGATCGCGAATTGCTTTATTTTAACGATGAAGCCACTTCTACACCAGTTCACATTATGTTCTACTTATAATTTCGATTACTTCCGTTGAGAAAGAGAGTAAAAAAAACAAAAAACAAAAAAAAAAAAAAATATGGAAAAATGGGATCTGAAAATACTGCATACATATGTTTAGATCATTTCTATATGGAATGTCGTCGTGTGTTTTTTATGACGCATTTAAATATTTTTGTAAAGATGAAATGTAAAGAAAGTGTGTCCAATTGCCAGATTATACATATGTAATATATCGGTTTTACGTAGAGCTACGTGCATGTGCATACACGGTGAAAAAAAAAATGATAAAAAATAAGTCAATTTGGCTGTAAATTATTCAAAATTTTCGCCTGTTGCCGGACGATTGAAATATATGTATGTACGTGTTTGATATACTGTCATTTTGATTTGCATTCAACTTGTACGTTTCGTTATATATTTGTTTTTATCTTTTTCCGAAATTTTGTTCAATGCATTTAACTATCGTATTCCCGAATTCA**GA**GAGTTTTCGAACGAACGAAATCGTGTCGAACGTCGCATGGAGTTTCAAAAATGCCGCTTTCGGGCCATGTTTCAGACAGCAATGGTCTCG**TA**CCTTcGACTGGATCACACAAGCAGGTTTATACAACTAGATCTATATCTAAGTGCAACCAAGTAAACTGCCATAATCAACCCGAACGTCATACAACACAACCTGCAATTATAACTAATTATAACTATTATCGGCCAAAAAAAAACAGAACAATAAGTCAACTCGTATCGTAATGCTAATGCTAATGCTATCGAATCCGAGCGAGTTCATTGAGCTAAACACCCATCTAATTAAACCAAAAAAAAAAACAATTCCGACCTATACTTCCTTAACCAGTTTATAAAGAACGTGTTTGATTTACTGCTAGTTATGTATTCTTAAATAGATCGCAAGCAATTCCCCGGCTACAAAGAGTTCTGGTACCCAAAACTAACTTAACTATCAATTACAATACAACTACTATGTACTTCCAATGAACCGAATTAATAATATTAACTGCGAAATGCCGTTCACCCCACTATAGTTTCTAACTAACCAACCAACCAAACAATCAATAATCTAATCCATATATACTCTGCAGTGCGTAATGCATATTATATTCTCTGCATTTTGCCACACACTTAACCCGATTTTTACTACCATAGCGATAAAGTTGAATTCCCCCCCCCTCCCTGTCAAAATAAACCAAACTAACCAAATACATAGCTATTTCAACTGCAGTTAGCCAAATTAACCTCCAGTTATATACTTATACCCGCCAGCGGACTCGTGAATTGTTGTTGAGTCTCGGCCTCGTTCTCGATATCTTGGCTAATATAGTACACTCCCTATACACTATATACACTGATAGAACAGTGCACCCACCTATCAATATCAATGTTCTGTCCCCGGTTAACTATCGCCATCAAAATGTATCATCGCTCCAGACTCAGAGACTTCAAGACTCGCAGACTCCCAGCGAATCAGGGCAAAATTCAGAGTTATTCAAGACCAGATGACGCTATGGCATCATCACCCAATGCGTACTCTATATATTTATTTGTCGCCACCGAATAAATCATTTTATATATGCTTGTATGCACACATATAATCTAACACATTTCCCTTCTTTTATATATATTTTTCCTTGACTTTTGACCTCATTGAAGGTTCCCGTCGAGGAGGCGCAATAAATATATACATATATATACAATATATGTGTTAAATTGCATTTCTGTTACTTCATTTCCAGTAAATGATTTCGACTTTCTGTTTATGCCCGTTTGTTGTGTTCTAAAAAACAAAAAAAAAAACAAAGCACTGGAATATTCTTGATTTTACGAATCTTTAAGTTATTCTTCTCTGTTGATTACACTCGTACAAAAACAATGGCGAAAGTATGATGTGAACTTTGATATTGTAATACTGAATACCAAAGCATACATGATCGTTTTAGCAATCAATCAATCCTGTTTTTTTGGTTTATCACGTTCTATTTAATTATTTTTTGTA**GT**GAATTCGC**AAAAGAACGAGAAA**AAGTAGAAAATAGACAAGAGTTTCTTAAACTTAGAAGGCAGCAGCAACTAGAAAGAGAGTTAAACGGCTATGTTGAATGGATTTGTAAAG**CTG**GTATAGTATTTACCCCTATTTCTTGTTTGTAAATCTATATATACACACACACACAATGACTAAACTCATGAATAACTCTCATACACACATATATACAAACATAAAAATTTAAACACTATGCGCCCCCATATGTTCATCCACATCCATATGTACATATATATATAAATTATAAAAACGACATTTATATCAGGTCCTTTTCTATGACTATAAAACTGAATTCAATGAAAATAGGCCAACTATAATCACATAAAATATTTTTTTAAAAATTCGAACAGGGATCACAAAAAATGCCTAACAAAAGATTCTTCTTTAATGTCATTGCTTCACTGAAAGATACATTAACTAAATATCGTTTATATCATTTTCATATAAAATGACGATATATTCATGCATATTTAAAGTGATACAAGAATAAAATATACGTTGTATTGAGATGTTCCCTAATAGCCAGCACACTTTTGGCACATTCTTAGCATCCCTCTCCTCATAGAATTGAAACCCTCGAACCTATATACATTGCCAAATTTGAAAGTGAATAAATTCCTCAATATACTCAGAATTAAAATAGTTTTCGTGGAAATATGGACATATATCCCTCATGCCGCCCCCAATATATATACATCTACCCACATTGACTATAATGTACATACGAGTATATATCCTTTCTGCTTATGTGTTGATCAAATCGATTCCCTTATTGTGACCCATCACCAATCACTCGATTTTAATGCTATTTCAGAGGAGGTAATCCTGGCCGAGGAGCGCACCACGGAAGAAGAGAAAATGCACATAATGGAgGGAAGCGGAgccaccaacttctccctgctgaagcaggccggcgacgtggaggagaaccccggccccatggcctcctccgaggacgtcatcaaggagttcatgcgcttcaaggtgcgcatggagggctccgtgaacggccacgagttcgagatcgagggcgagggcgagggccgcccctacgagggcacccagaccgccaagctgaaggtgaccaagggcggccccctgcccttcgcctgggacatcctgtcccctcagttccagtacggctccaaggcctacgtgaagcaccccgccgacatccccgactacttgaagctgtccttccccgagggcttcaagtgggagcgcgtgatgaacttcgaggacggcggcgtggtgaccgtgacccaggactcctccctgcaggacggcgagttcatctacaaggtgaagctgcgcggcaccaacttcccctccgacggccccgtaatgcagaagaagaccatgggctgggaggcctccaccgagcggatgtaccccgaggacggcgccctgaagggcgagatcaagatgaggctgaagctgaaggacggcggccactacgacgccgaggtcaagaccacctacatggccaagaagcccgtgcagctgcccggcgcctacaagaccgacatcaagctggacatcacctcccacaacgaggactacaccatcgtggaacagtacgagcgcgccgagggccgccactccaccggcgcctaa**AA**GGAAGCGGAgccaccaacttctccctgctgaagcaggccggcgacgtggaggagaaccccggccccAtggtgagcaagggcgaggagctgttcaccggggtggtgcccatcctggtcgagctggacggcgacgtaaacggccacaagttcagcgtgtccggcgagggcgagggcgatgccacctacggcaagctgaccctgaagttcatctgcaccaccggcaagctgcccgtgccctggcccaccctcgtgaccaccctgacctacggcgtgcagtgcttcagccgctaccccgaccacatgaagcagcacgacttcttcaagtccgccatgcccgaaggctacgtccaggagcgcaccatcttcttcaaggacgacggcaactacaagacccgcgccgaggtgaagttcgagggcgacaccctggtgaaccgcatcgagctgaagggcatcgacttcaaggaggacggcaacatcctggggcacaagctggagtacaactacaacagccacaacgtctatatcatggccgacaagcagaagaacggcatcaaggtgaacttcaagatccgccacaacatcgaggacggcagcgtgcagctcgccgaccactaccagcagaacacccccatcggcgacggccccgtgctgctgcccgacaaccactacctgagcacccagtccgccctgagcaaagaccccaacgagaagcgcgatcacatggtcctgctggagttcgtgaccgccgccgggatcactctcggcatggacgagctgtacaagtaa**ACCGGT**

**D. cac_5_6_GFP_mRFP1**

**GCGGCCGC**caaaATGttGGAAAAAACGGAGGCCTATTTTTTATGCATTTTCTGTGTAGAAGCGTCGCTCAAGATCCTCGCCTTAGGGCTTGTTCTGCATAAACACTCCTATCTCAGGAATATTTGGAACATCATGGATTTTTTCGTTGTAGTTACGGGGTAAATAATGAACCAAACTATTTTCGAAACATATGCACCAATTTTATTTTCAACTAATATCGTATTAAAAAAAAAACTACACGCCGTGCAAGCATTATCTGTTTTTATTCAAACCAAACCAATATCACCTATGTTTAAGTCTTGATCATAATGAATCCCAAGAAAAAGTTAACTTAATATTCTCAGAAAAGCTTAGTTACTCTTTTTCTAGATTCATTACAGATCTAATCTAACAATTAGACATGTCATTTTAGTTGTAAGATGTAAAATTGTACAAATCACAAACTTTATATTAAGATATGTATTTAGTTATTAAGTGCCCAATATATTTTTGTGTTGCGGTAAGATATTTGCCTCAACTAAATCATATAAATGTATTATAAGTAAGCGACTATATTAACAACTTCTCCCATATATGTTTAAAATCGGGTAACAAACTCGAAATGGCAAATAACAATGCCAATTGCAGAGCCATGACGATATTTGCTGAGGCCAATATAGATGTTGACCTGCGTATGTTACGATCTTTTCGTGTTTTACGCCCACTGAAGCTCGTATCCCGAATTCCAAGTAAGACATCTGCCAACAAAAAGAACCATTAGTTGCTACTACCAAAAACCTAGGCCAGATAAAAAAAACATCGTTTTCTTCAGTAAACATATATATTAAACTAAACAAATTACACTCGAAAAAGCTAGTTGTCCTATATGGATACTTACTATAAATTTAATTGGATTAGGAGTAGCGAAAACGGCATAAACAGAAAACAACCTTAGAGGCTAGGAGCCACGAAAATTAGATGCTAAAATATATTTATTAGTTATATATTAAGTAGCTACATATAATATAAATTTATAGCTCAATCAGGCTTCCGATTTCCTTTGATTCTTCAAATGAAACAAAATAAATTTTATTAAGGAAAACGGTAGCACGTTTGTAAAATTCTCTTATAAACAAAATAGGATTAGTATTATTCAACTATAGATGGCATCAGTAGGATGCAATGTGTTATCGTGTAGGTTTATAATATAAATCAATTCATGTACAGTGGTAGCCACGAATTTAGGAACTTTTAAAGCGTATTATCTTTCGTGGCTATCACTCGGTAGTTTTTATGAATATATATATATATACATATAGCTGTATATTCTTTCTGATTTGTAGTGATTTCGACTAGCCATTTTTATTTATGTTTACAAAATATTATATTAGCATTATAATTACTAGATATATATATACATAAGTGAGTCAAAGACTATATAACTGTGTGTGATTCTCGCGACTATAATGTTTATAAATTAGACGTTAAGTCTTGACCGTAATTCAAGTTACCTTTCCGTTCGATTTAAAATAGATCGCAACTTGCAAGTCATCGATGAATCCCCTTTTAAAGGTGGTAGAATAATAGCGAATATTATTGATTTTCGATAATAGCTGGCACATAAATGATAAATTTACATCTATATATAATGCCGAAACTATAGAGTGACTGACCAAAATCACTTTTAGCCAATTGTAGATGTTTTCATTTATTTTTCATCGGAAATTTGTATCTCTTAAAGTGAAAATTTACTTTTACTCTCCTAGAACTTTGAAATAAGCAGTGTGTATGTTATAAGCAATAGATTCATGTTATATTTTAAATTATAGCCTTAACCTATATTCAATTCCATAGCTGGACCAAAACCATAGTTTACATTTGCTCTTCAACATTAATGCCTGAAAATATTGATCAATATTGTTCGGAATGAAGAGCAAAGCTAAACTATTCTGCGTTTTCGCTTGGGTTACCCATTGTGTGTGCGAAAAATATGAACTAACCGCGCGTTAAACGTTATTATTATTAAGTATTATTTAAGTTATTTAAATTTGTACACTCCTTAGTTGCTTCTTGCTTTCGATTCAAGTTTCGATTTAAGCTGCACAATACATATGTATAAACTTTTGTTTTCAACGTACTCATATGATTTCGATATTCCAATTATGATTATCCGATCTGTATCCATTGAGTATCGCTAATCAATATATAACTTCATTTCGATCGCTTAACGTCTGCAAACAGATTCATGACACAGTACCCACAAATAGGGCCCGAGGTAGACCTAAGAACACTTAGAGCCATTCGTGTGCTACGGCCCTTAAAATTaaAGTGTCTGGAATTCCTAGTGAGTAGTTCCTCTGTTTAATTCTACATGTCGTTGTTGTCTTTCCATTCTTGATTTTTAAAGCCAAACCTCACTCCAGACTCTTTAATGTGCGTAAATGAGATTGTTTTTTTTTTTAAATTGTGTTTTAAGTTCTAGTACGAGTGAGTGGAAAACGAGTGAGAAAAGAGCCTGCATTTTTGCCACCACTTAGTTTTCGATTTTCGTTTGAAACGTACTCTTTTTGACTAGAATGATTGTTTGTGGCACATGATTGTCGTGGAGGAGCTTTCAATGTCTGCGACAATCATGTCCTAAATGTGAAACACATACCCAGCATATGCATCTTTCTAATCCAACGTCCATCTTCTCCAAACAACTAACCAATCAACTTAACTGAATCGTTTCTGGATCGAATCGGAACGGCAACGTCAACAGGTTTACAAGTAcTTTTAcAATCTATATTCAAGGCGATGGCACCTTTACTGCAAATCGGTCTCTTGGTGTTGTTTGCAATCGTATTTTTTGCAATCATTGGACTCGAGTTTTATTCGGGCGCACTGCATAAGACTTGTTATAGCTCAGAAGATCCAAGGAAGCGGAgccaccaacttctccctgctgaagcaggccggcgacgtggaggagaaccccggccccatggtgagcaagggcgaggagctgttcaccggggtggtgcccatcctggtcgagctggacggcgacgtaaacggccacaagttcagcgtgtccggcgagggcgagggcgatgccacctacggcaagctgaccctgaagttcatctgcaccaccggcaagctgcccgtgccctggcccaccctcgtgaccaccctgacctacggcgtgcagtgcttcagccgctaccccgaccacatgaagcagcacgacttcttcaagtccgccatgcccgaaggctacgtccaggagcgcaccatcttcttcaaggacgacggcaactacaagacccgcgccgaggtgaagttcgagggcgacaccctggtgaaccgcatcgagctgaagggcatcgacttcaaggaggacggcaacatcctggggcacaagctggagtacaactacaacagccacaacgtctatatcatggccgacaagcagaagaacggcatcaaggtgaacttcaagatccgccacaacatcgaggacggcagcgtgcagctcgccgaccactaccagcagaacacccccatcggcgacggccccgtgctgctgcccgacaaccactacctgagcacccagtccgccctgagcaaagaccccaacgagaagcgcgatcacatggtcctgctggagttcgtgaccgccgccgggatcactctcggcatggacgagctgtacaagtaa**AA**GGAAGCGGAgccaccaacttctccctgctgaagcaggccggcgacgtggaggagaaccccggccccAtggcctcctccgaggacgtcatcaaggagttcatgcgcttcaaggtgcgcatggagggctccgtgaacggccacgagttcgagatcgagggcgagggcgagggccgcccctacgagggcacccagaccgccaagctgaaggtgaccaagggcggccccctgcccttcgcctgggacatcctgtcccctcagttccagtacggctccaaggcctacgtgaagcaccccgccgacatccccgactacttgaagctgtccttccccgagggcttcaagtgggagcgcgtgatgaacttcgaggacggcggcgtggtgaccgtgacccaggactcctccctgcaggacggcgagttcatctacaaggtgaagctgcgcggcaccaacttcccctccgacggccccgtaatgcagaagaagaccatgggctgggaggcctccaccgagcggatgtaccccgaggacggcgccctgaagggcgagatcaagatgaggctgaagctgaaggacggcggccactacgacgccgaggtcaagaccacctacatggccaagaagcccgtgcagctgcccggcgcctacaagaccgacatcaagctggacatcacctcccacaacgaggactacaccatcgtggaacagtacgagcgcgccgagggccgccactccaccggcgccTaa**ACCGGT**

**E. cac_34_GFP_mRFP1**

**GCGGCCGC**caaaATGAGGCACAATGGCTCTCCACTGGCCAGATCTCCGAGTCCTCGACGGCGTGGCCATCAATACATACATCATGATATCGGGTTCTCCGATACCGTATCTAATGTTGTAGAGATGGTCAAGGAGACTCGTCATCCTAGGCATGGCAACAGTCATCCGCGGTATCCAAGAGGTATTTATTGGCTCGACATCCATGGCATTTAATCATTAACTACTTTCAACTGACTATCGTGTGTGTGTGTGTGTGTGCTGGTTGTAAGCCAAATGATCTGGTTAGGGATTCGTCAACATTATCCTGTTATCGAGTTGTCTGTCCTTGATGTTGCCCTTAATCACTTCGTATCCATATGAGATCGGCTTGCAAGGGGCACACACAGAAAACAAAGGGACTTGGACATTCCAGGGATTTTTGATCTATTTTGTAGAACCGTAGTTATATACATATATACATATATGTCTACAATTCCATAGCTATTAACGAAATCTTTTAATACAAATATATTATATTTCAATAATAATTTTATCGTTTCCGTTAAGTCGGTCCTCTATTGTCGTTTATCGTTTATCTCTTCGAAAGCAATTTGAAAATCGCAAAAAACCAAAATGAAAAAAAAAAAAACAAAACAAAACAAAACAAAAGAAGAGAAGAGAAAATATGTTGCTAAGATCCAATACGTAAAATGTTGTGCCCCGACATCCTTAATTATTACTTTTTTTTATTATTGTTGTTATATGTACAATATAGGTTCATGGTCAGCATCGACAAGTCCGGCCCGTTCGCCTTCGCCTTCTCGATATGGTGGTCATTTGTCTCGCAGTAAACGCACTCAACTGCCTTATCCCACATATGGGACAACCAGTCTATGTCAAAGATCACGATCGCCGAGTCCCGCTAGACTTCAGGAGATGCGTGAACGAGACAGACTTGGTTttATGGGATTGATATGGGTACATCCTTAGCACTTGTTATCTAAAGTCCAACAAGCTCAATCAAAAATCAACTTTAAACAATTTAACAAAAACAACTACTCAACTTGTTTTCTATATATGCTAGTATTTTCTTCTACACAAATTTCGACTTCAACCATCTCAATTTTAACAAACATCAAAGATCAGACATGCTTACTCGTAAATCGTTAACTCGTAACTCGTATCTCGTAACTAACTTGTATTCGTACATAGTTGCCAACCATGCTTAACATGTATACATGTATTGTCATGCCCAGACGTTCAATCAGAAAACAAACAGTCAATCAATCAATCAATCAATCAATCAATCAATCAGCCAATCAGTCAACCATAAGCTTCAACAAATCGAATAGTTCAAATACTCTTTTCATACATCCAAACACAGCTCTCAAAAATTCATTCAGATCTGCGATCTACCACACCGAAGAACTCATGATTATGTGACCAACTATTCGTATTTCGTATTCGTACTCTTGTTACACTAGCAGCGTTTATGTTTTCATTTGTCTAGAAGCCATCCTCGTAACATCCACCTATATTATAATCACATCTCGTATGTATGGTGCTGACATGTACAGACATATTCATCCTTGTCATTAAACTAACACACTGAATCTCTGGCAAATGAACTGTTGAATAGCAATAGAGGAGTTTAAATATATACGATTTTTATGTCAGGACTACTTTCGACGGACTCTTAGTAGAACACCAAGTACTGGTCACTTTTTAAAAACAGCATCGATGTGAGAGAAATGTGAATATATTATGGAACAGCAATGTGTAGGGGTGAGATAAGTGAACATCATTTCCTAGTCTATTCCACAAAGAAGGTGAAGTTCGTAAGCCATAGTATGTAGAACATATATATATATATATAAATTTTTTTTTTTGTTCAAAATTGACCTCTGTCAGCCTGACCAGCACAGGATAAGACACTGACCAAGAATCTCTATTTAAAGCTTGCTTCTTGTTCCAAAAAAAACACAAAAAAAAAAACACCCAGTTACTACCACAGATATGCAGGCAATGTTCCAGTATTGTTTTTTGTACCAGTTTTGGGCGGTGAAATCGACTGAGCTACATACATACTCGTCTATGTAATATTATAACCAGAGGAATAATTTGGGTTGCAGCCTTCGGGTGTTTCAGTATCTAGACGTGTGTGCATATTTATCTATCTTATCTATCTGTGAATGTGCGTCAATAGTATTCTGCACCATTTCTTAATCCATCCTTTTCGTTTTTGCATAATTGATATCTCTATAGATCAAATCGAAATCAAAACAGTCACGCATGTTGCCATGAACAATTTAGCTGCCGACCGGTCATTACCCCAAATCATCCAACTACTTGTGTATATTTCTCTCAATACATTATATATAGCACTGTTGTTAATGTTTCATATTTTTCTAAGTTGTATAATTCTGTAGGGTGCTCAAATAATTCCACACGCTTATGAACGTAGATACAGTGAATGCATTTTTGGCATAGCAACAATTTTTAAACAACATTAAAGTTTAATATGTAAACTACGTTCTGATTGGGACATGTTCATCCTTTACACAATATCGTAAAATCACCATTAAATCGTAATTGTGCAAAAAATATTAACTTCGAGTTTTGAAAGAACCTGTCAAATAAATCTTGTTTCTATAAAGTTTGTATGTGTATTTTTAGAGATAACTAATGAAATCATAAATCGTTTAACGCTAACTTTGACCTGTACTTTCAGAATTCAATAGTCCTTACTGAGACTTAACAAAATCCCACTAAATGTCGCACAATAAAATTCAACATAAATCTGGTTACGATAAAGTCTGTCTGATTGTCTTCAGTTATTAGATAGTTAGTCCTAATCTAATCCAAATGAACATGTCCCATTCTATATTATGTTTCATATATTTGTATGTATGTTTTAAGTCCATGTTAATGTCAATGAAGAACGACTTAGGTACACTTTGTTCAGTATCATAATAGCAGAGTATTTTTGCAACGACTTATGCCCTAAACTGCACTTGTTCAGCACACAGGGAAGTAATTTTTCGATTTCCCGCAGGTGTATCGCATGTACAGCATAGTTACCCAACACTGGCCTCCCGAAGAGCCGGAATCGGAAGACGCCTTCCTCCGACTCCCAGTAAACCGTCAACACTGCAGCTCAAGCCAACCAATATCAATTTCCCGAAGCTCAATGCCAGTCCCACACATAGGAAGCGGAgccaccaacttctccctgctgaagcaggccggcgacgtggaggagaaccccggccccatggtgagcaagggcgaggagctgttcaccggggtggtgcccatcctggtcgagctggacggcgacgtaaacggccacaagttcagcgtgtccggcgagggcgagggcgatgccacctacggcaagctgaccctgaagttcatctgcaccaccggcaagctgcccgtgccctggcccaccctcgtgaccaccctgacctacggcgtgcagtgcttcagccgctaccccgaccacatgaagcagcacgacttcttcaagtccgccatgcccgaaggctacgtccaggagcgcaccatcttcttcaaggacgacggcaactacaagacccgcgccgaggtgaagttcgagggcgacaccctggtgaaccgcatcgagctgaagggcatcgacttcaaggaggacggcaacatcctggggcacaagctggagtacaactacaacagccacaacgtctatatcatggccgacaagcagaagaacggcatcaaggtgaacttcaagatccgccacaacatcgaggacggcagcgtgcagctcgccgaccactaccagcagaacacccccatcggcgacggccccgtgctgctgcccgacaaccactacctgagcacccagtccgccctgagcaaagaccccaacgagaagcgcgatcacatggtcctgctggagttcgtgaccgccgccgggatcactctcggcatggacgagctgtacaagtaaAAGGAAGCGGAgccaccaacttctccctgctgaagcaggccggcgacgtggaggagaaccccggccccAtggcctcctccgaggacgtcatcaaggagttcatgcgcttcaaggtgcgcatggagggctccgtgaacggccacgagttcgagatcgagggcgagggcgagggccgcccctacgagggcacccagaccgccaagctgaaggtgaccaagggcggccccctgcccttcgcctgggacatcctgtcccctcagttccagtacggctccaaggcctacgtgaagcaccccgccgacatccccgactacttgaagctgtccttccccgagggcttcaagtgggagcgcgtgatgaacttcgaggacggcggcgtggtgaccgtgacccaggactcctccctgcaggacggcgagttcatctacaaggtgaagctgcgcggcaccaacttcccctccgacggccccgtaatgcagaagaagaccatgggctgggaggcctccaccgagcggatgtaccccgaggacggcgccctgaagggcgagatcaagatgaggctgaagctgaaggacggcggccactacgacgccgaggtcaagaccacctacatggccaagaagcccgtgcagctgcccggcgcctacaagaccgacatcaagctggacatcacctcccacaacgaggactacaccatcgtggaacagtacgagcgcgccgagggccgccactccaccggcgccTaa**ACCGGT**

**F. cac_34_GFP_mRFP1_remade**

**GCGGCCGC**caaaATGAGGCACAATGGCTCTCCACTGGCCAGATCTCCGAGTCCTCGACGGCGTGGCCATCAATACATACATCATGATATCGGGTTCTCCGATACCGTATCTAATGTTGTAGAGATGGTCAAGGAGACTCGTCATCCTAGGCATGGCAACAGTCATCCGCGGTATCCAAGAGGTATTTATTGGCTCGACATCCATGGCATTTAATCATTAACTACTTTCAACTGACTATCGTGTGTGTGTGTGTGTGTGCTGGTTGTAAGCCAAATGATCTGGTTAGGGATTCGTCAACATTATCCTGTTATCGAGTTGTCTGTCCTTGATGTTGCCCTTAATCACTTCGTATCCATATGAGATCGGCTTGCAAGGGGCACACACAGAAAACAAAGGGACTTGGACATTCCAGGGATTTTTGATCTATTTTGTAGAACCGTAGTTATATACATATATACATATATGTCTACAATTCCATAGCTATTAACGAAATCTTTTAATACAAATATATTATATTTCAATAATAATTTTATCGTTTCCGTTAAGTCGGTCCTCTATTGTCGTTTATCGTTTATCTCTTCGAAAGCAATTTGAAAATCGCAAAAAACCAAAATGAAAAAAAAAAAAACAAAACAAAACAAAACAAAAGAAGAGAAGAGAAAATATGTTGCTAAGATCCAATACGTAAAATGTTGTGCCCCGACATCCTTAATTATTACTTTTTTTTATTATTGTTGTTATATGTACAATATAGGTTCATGGTCAGCATCGACAAGTCCGGCCCGTTCGCCTTCGCCTTCTCGATATGGTGGTCATTTGTCTCGCAGTAAACGCACTCAACTGCCTTATCCCACATATGGGACAACCAGTCTATGTCAAAGATCACGATCGCCGAGTCCCGCTAGACTTCAGGAGATGCGTGAACGAGACAGACTTGGTTttATGGGATTGATATGGGTACATCCTTAGCACTTGTTATCTAAAGTCCAACAAGCTCAATCAAAAATCAACTTTAAACAATTTAACAAAAACAACTACTCAACTTGTTTTCTATATATGCTAGTATTTTCTTCTACACAAATTTCGACTTCAACCATCTCAATTTTAACAAACATCAAAGATCAGACATGCTTACTCGTAAATCGTTAACTCGTAACTCGTATCTCGTAACTAACTTGTATTCGTACATAGTTGCCAACCATGCTTAACATGTATACATGTATTGTCATGCCCAGACGTTCAATCAGAAAACAAACAGTCAATCAATCAATCAATCAATCAATCAATCAATCAGCCAATCAGTCAACCATAAGCTTCAACAAATCGAATAGTTCAAATACTCTTTTCATACATCCAAACACAGCTCTCAAAAATTCATTCAGATCTGCGATCTACCACACCGAAGAACTCATGATTATGTGACCAACTATTCGTATTTCGTATTCGTACTCTTGTTACACTAGCAGCGTTTATGTTTTCATTTGTCTAGAAGCCATCCTCGTAACATCCACCTATATTATAATCACATCTCGTATGTATGGTGCTGACATGTACAGACATATTCATCCTTGTCATTAAACTAACACACTGAATCTCTGGCAAATGAACTGTTGAATAGCAATAGAGGAGTTTAAATATATACGATTTTTATGTCAGGACTACTTTCGACGGACTCTTAGTAGAACACCAAGTACTGGTCACTTTTTAAAAACAGCATCGATGTGAGAGAAATGTGAATATATTATGGAACAGCAATGTGTAGGGGTGAGATAAGTGAACATCATTTCCTAGTCTATTCCACAAAGAAGGTGAAGTTCGTAAGCCATAGTATGTAGAACATATATATATATATATAAATTTTTTTTTTTGTTCAAAATTGACCTCTGTCAGCCTGACCAGCACAGGATAAGACACTGACCAAGAATCTCTATTTAAAGCTTGCTTCTTGTTCCAAAAAAAACACAAAAAAAAAAACACCCAGTTACTACCACAGATATGCAGGCAATGTTCCAGTATTGTTTTTTGTACCAGTTTTGGGCGGTGAAATCGACTGAGCTACATACATACTCGTCTATGTAATATTATAACCAGAGGAATAATTTGGGTTGCAGCCTTCGGGTGTTTCAGTATCTAGACGTGTGTGCATATTTATCTATCTTATCTATCTGTGAATGTGCGTCAATAGTATTCTGCACCATTTCTTAATCCATCCTTTTCGTTTTTGCATAATTGATATCTCTATAGATCAAATCGAAATCAAAACAGTCACGCATGTTGCCATGAACAATTTAGCTGCCGACCGGTCATTACCCCAAATCATCCAACTACTTGTGTATATTTCTCTCAATACATTATATATAGCACTGTTGTTAATGTTTCATATTTTTCTAAGTTGTATAATTCTGTAGGGTGCTCAAATAATTCCACACGCTTATGAACGTAGATACAGTGAATGCATTTTTGGCATAGCAACAATTTTTAAACAACATTAAAGTTTAATATGTAAACTACGTTCTGATTGGGACATGTTCATCCTTTACACAATATCGTAAAATCACCATTAAATCGTAATTGTGCAAAAAATATTAACTTCGAGTTTTGAAAGAACCTGTCAAATAAATCTTGTTTCTATAAAGTTTGTATGTGTATTTTTAGAGATAACTAATGAAATCATAAATCGTTTAACGCTAACTTTGACCTGTACTTTCAGAATTCAATAGTCCTTACTGAGACTTAACAAAATCCCACTAAATGTCGCACAATAAAATTCAACATAAATCTGGTTACGATAAAGTCTGTCTGATTGTCTTCAGTTATTAGATAGTTAGTCCTAATCTAATCCAAATGAACATGTCCCATTCTATATTATGTTTCATATATTTGTATGTATGTTTTAAGTCCATGTTAATGTCAATGAAGAACGACTTAGGTACACTTTGTTCAGTATCATAATAGCAGAGTATTTTTGCAACGACTTATGCCCTAAACTGCACTTGTTCAGCACACAGGGAAGTAATTTTTCGATTTCCCGCAGGTGTACCGCATGTACAGCATAGTTACCCAACACTGGCCTCCCGAAGAGCCGGAATCGGAAGACGCCTTCCTCCGACTCCCAGTAAACCGTCAACACTGCAGCTCAAGCCAACCAATATCAATTTCCCGAAGCTCAATGCCAGTCCCACACATAGGAAGCGGAgccaccaacttctccctgctgaagcaggccggcgacgtggaggagaaccccggccccatggtgagcaagggcgaggagctgttcaccggggtggtgcccatcctggtcgagctggacggcgacgtaaacggccacaagttcagcgtgtccggcgagggcgagggcgatgccacctacggcaagctgaccctgaagttcatctgcaccaccggcaagctgcccgtgccctggcccaccctcgtgaccaccctgacctacggcgtgcagtgcttcagccgctaccccgaccacatgaagcagcacgacttcttcaagtccgccatgcccgaaggctacgtccaggagcgcaccatcttcttcaaggacgacggcaactacaagacccgcgccgaggtgaagttcgagggcgacaccctggtgaaccgcatcgagctgaagggcatcgacttcaaggaggacggcaacatcctggggcacaagctggagtacaactacaacagccacaacgtctatatcatggccgacaagcagaagaacggcatcaaggtgaacttcaagatccgccacaacatcgaggacggcagcgtgcagctcgccgaccactaccagcagaacacccccatcggcgacggccccgtgctgctgcccgacaaccactacctgagcacccagtccgccctgagcaaagaccccaacgagaagcgcgatcacatggtcctgctggagttcgtgaccgccgccgggatcactctcggcatggacgagctgtacaagtaaAAGGAAGCGGAgccaccaacttctccctgctgaagcaggccggcgacgtggaggagaaccccggccccAtggcctcctccgaggacgtcatcaaggagttcatgcgcttcaaggtgcgcatggagggctccgtgaacggccacgagttcgagatcgagggcgagggcgagggccgcccctacgagggcacccagaccgccaagctgaaggtgaccaagggcggccccctgcccttcgcctgggacatcctgtcccctcagttccagtacggctccaaggcctacgtgaagcaccccgccgacatccccgactacttgaagctgtccttccccgagggcttcaagtgggagcgcgtgatgaacttcgaggacggcggcgtggtgaccgtgacccaggactcctccctgcaggacggcgagttcatctacaaggtgaagctgcgcggcaccaacttcccctccgacggccccgtaatgcagaagaagaccatgggctgggaggcctccaccgagcggatgtaccccgaggacggcgccctgaagggcgagatcaagatgaggctgaagctgaaggacggcggccactacgacgccgaggtcaagaccacctacatggccaagaagcccgtgcagctgcccggcgcctacaagaccgacatcaagctggacatcacctcccacaacgaggactacaccatcgtggaacagtacgagcgcgccgagggccgccactccaccggcgccTaa**ACCGGT**
