# Supplementary figures and images for: Bichromatic Exon-Reporters Reveal Voltage-Gated Ca2+-Channel Splice–Isoform Diversity across Drosophila Neurons In Vivo
Source: eNeuro. 2025 Aug 14;12(8):ENEURO.0582-24.2025. doi: 10.1523/ENEURO.0582-24.2025 (PMC12370356; doi:10.1523/ENEURO.0582-24.2025)

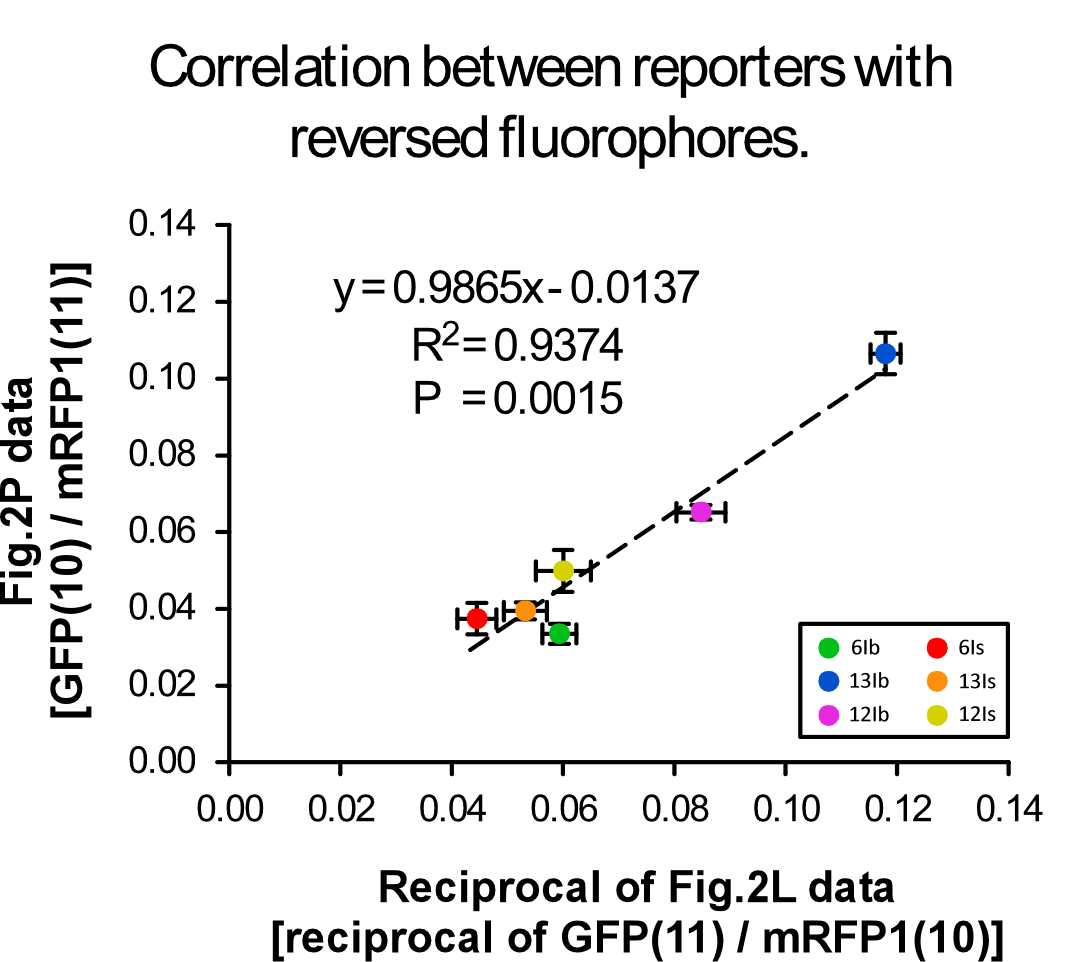

Supplement: Figure 2-1 — Correlation between two versions of an exon 10 versus 11 reporter with fluorophore construct order reversed. Terminal ratios obtained from exon reporter GFP (10) / mRFP1 (11) (shown in Fig. 2P) are plotted against the inverse of terminal ratios from exon reporter mRFP1 (10) / GFP (11) (shown in Fig. 2L). Pearson’s correlation coefficient indicated a significant correlation (R2 = 0.937, P = 0.0015). Download Figure 2-1, TIF file. [file eneuro-12-ENEURO.0582-24.2025-s003.tif]

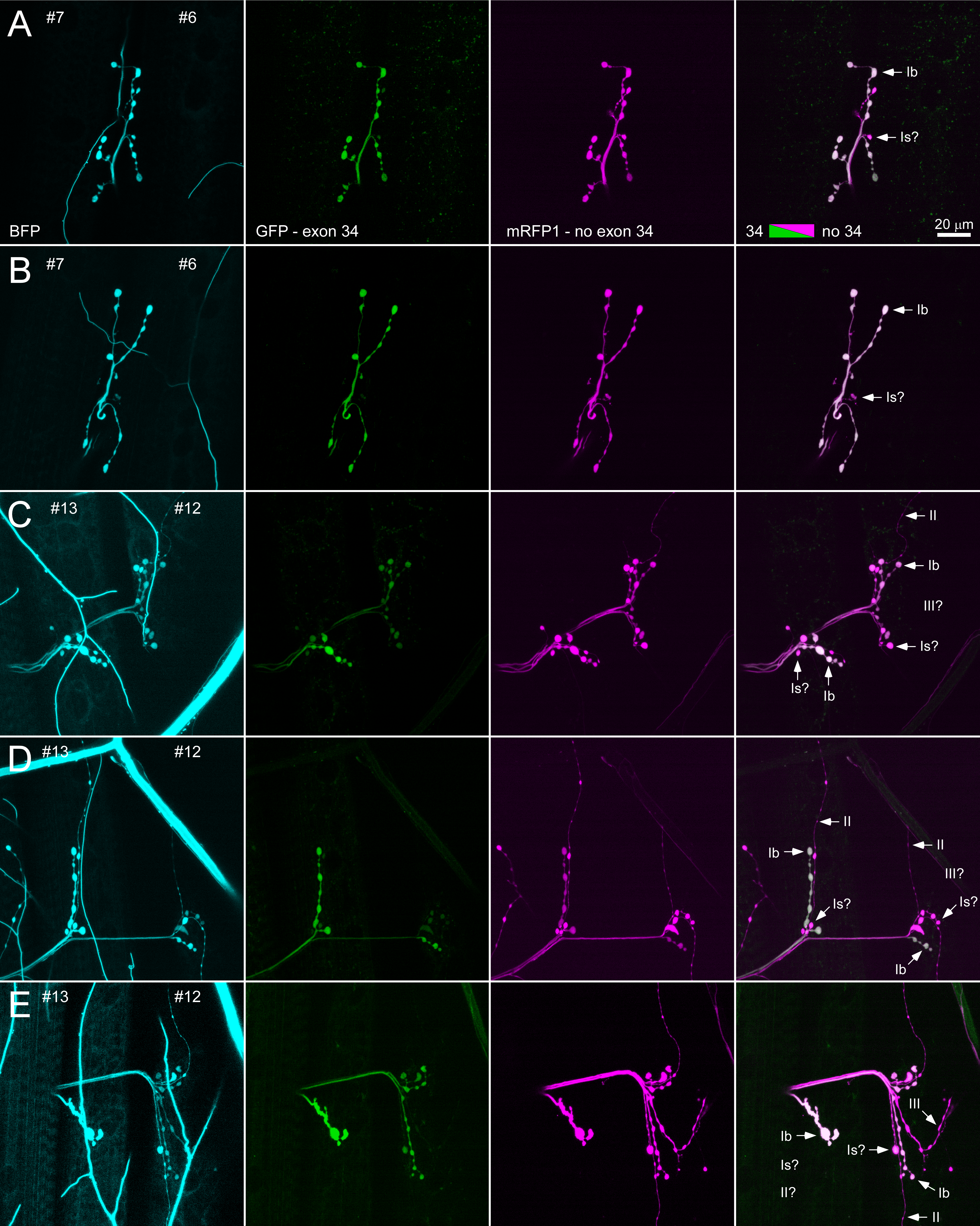

Supplement: Figure 4-1 — Type-Is terminals missing phenotype observed when expressing the exon 34 reporter. A-B. Muscle fibers #6 and #7 showing the TagBFP expression control, along with GFP and mRFP1 of the exon 34 reporter when expressed pan-neuronally using the nSyb-GAL4 driver. Type-Ib terminals can be clearly identified, while type-Is terminals appear “vestigial” in some cases (A), if not missing (B). This phenotype was observed in two of the 9 preparations examined. C-E. Muscle fibers #13 and #12 showing the TagBFP expression control, along with the exon 34 reporter, when expressed with the nSyb-GAL4 driver. An attempt is made to identify each terminal type, but that cannot be done definitively when one terminal is missing (C & D: type III missing), or more than one terminal is missing (E: type-II and type III missing), on one or both muscle fibers. Download Figure 4-1, TIF file. [file eneuro-12-ENEURO.0582-24.2025-s004.tif]
